# Gene expression profiles in canine mammary carcinomas of various grades of malignancy

**DOI:** 10.1186/1746-6148-9-78

**Published:** 2013-04-15

**Authors:** Karol M Pawłowski, Henryk Maciejewski, Izabella Dolka, Jan A Mol, Tomasz Motyl, Magdalena Król

**Affiliations:** 1 Department of Physiological Sciences, Faculty of Veterinary Medicine, Warsaw University of Life Sciences - WULS, Nowoursynowska 159, Warsaw, 02-776, Poland; 2 Institute of Computer Engineering, Control and Robotics I-6, Wroclaw University of Technology, Wybrzeże Wyspiańskiego 27, Wroclaw, 50-320, Poland; 3 Department of Pathology and Veterinary Diagnostics, Faculty of Veterinary Medicine, Warsaw University of Life Sciences - WULS, Nowoursynowska 159, Warsaw, 02-776, Poland; 4 Department Clinical Sciences of Companion Animals, Faculty of Veterinary Medicine, Utrecht University, Yalelaan 108, Utrecht, 3584 CM, The Netherlands

## Abstract

**Background:**

The frequency of mammary malignancies in canine patients is even three times over than in human. In various types of cancer different intracellular signalling pathways are perturbed, thus the patients with pathologically the same type of cancer often have dissimilar genetic defects in their tumours and respond in a heterogeneous manner to anticancer treatment. That is why the objective of the hereby study was to assess the gene expression profiles in canine mammary carcinomas (in unsupervised manner) classified by pathologists as grade 1 (well differentiated), grade 2 (moderately differentiated) and grade 3 (poorly differentiated) and compare their molecular and pathological classifications.

**Results:**

Our unsupervised analysis classified the examined tissues into three groups. The first one significantly differed from the others and consisted of four carcinomas of grade 3 and one carcinoma of grade 2. The second group consisted of four grade 1 carcinomas. The very heterogeneous (based on their pathological parameters) group was the last one which consisted of two grade 1 carcinomas, two grade 3 carcinomas and five grade 2 carcinomas. Hierarchical dendrogram showed that the most malignant tumour group had significantly distinct gene expression.

**Conclusions:**

Molecular classification of canine mammary tumours is not identical with pathological classification. In our opinion molecular and pathological characterization of canine mammary malignancy can complement one another. However, furthers studies in this field are required.

## Background

The frequency of malignancies such as breast cancer has been constantly increasing. This pathological tendency has now become the most common form of malignancy among women in almost the whole Europe and North America continents. In bitches the incidence of mammary tumour has been found to be at the level of even three times more frequent than in human [1]. About 50% of all mammary tumours are malignant [2]. The aetiology of breast cancer is very complex and not completely comprehend. Genetic, hormonal, dietary, environmental and carcinogenic factors are known as being mediators of tumourigenesis in human and canine [1-3].

Although extensive research on breast cancer has been in the process for over decades, yet still, challenges prevail in early diagnosis and management of cancer patients. Furthermore, molecular mechanisms underlying breast cancer progression remain poorly understood [4]. This deficit has led to an increased interest in diverse and complex molecular biology of the malignancy. Investigators are focused on discovering novel predictive markers in mammary cancer. Such bio-markers play the pivotal role in terms of efficiency in patients management. The conventional approach to cancer therapy provide treatment according to actual organ or tissue in which the cancer originates. However, different intracellular signalling pathways are perturbed in various types of cancer. Thus, the patients with the same type of cancer often have dissimilar genetic defects in their tumours and respond in a heterogeneous manner to anticancer treatment [4].

That is why the objective of this study had been targeted to assess gene expression profiles of canine mammary carcinoma (in unsupervised manner) diagnosed by pathologists as grade 1 (well differentiated), grade 2 (moderately differentiated) or grade 3 (poorly differentiated) and to be compared against their molecular and pathological classifications. The molecular classification reflects biological processes and pathways within the tumour cell, not only the morphological features. It is also helpful in better understanding of cancer biology (to find genes and pathways responsible for tumourigenesis or to explore molecular interactions within the tumour). Our analysis have exposed, that some pathologically distinct tumours may have similar gene expression and vice versa. Probably that is why sometimes patients with similar pathological type of cancer have various outcome and respond in a heterogeneous manner to anticancer agents. Despite there is a significant correlation between pathological diagnosis and prognosis in canine patients with mammary tumour, there is still a need to improve treatment strategies [5,6]. Therefore, the molecular interaction within the tumour should be kept explored. That is why in our opinion pathological and molecular examination could significantly complement one another.

## Methods

### Tissue samples

Tumour samples of canine mammary cancers were obtained from patients subjected to surgery. The tumours then, were divided into two halves, one of them was fixed in 10% neutral buffered formalin and routinely embedded in paraffin to perform histological examination. The other part was snap frozen in liquid nitrogen and stored in −80°C. Four μm samples from paraffin blocks were placed onto glass slides, stained with haematoxylin – eosin (HE) and examined by certified pathologists (Prof. Dr. hab. Elżbieta Malicka and Dr. Izabella Dolka, both from the Warsaw University of Life Sciences, Poland). The tumour types of specimens had been classified based on the World Health Organization (WHO) Histological Classification and Mammary Tumours of the Dog and Cat classification [2]. Histological tumour grading was conducted on HE-stained sections using a Misdorp classification [2]. The mammary carcinoma grading was assessed in respect to tubule formation, degree of differentiation and mitotic index. All the tumours examined were classified as grade 1, grade 2 and grade 3 (6 tumours in each group). Unfortunately survival data of these dogs was unavailable.

### Microarray analysis

The total RNA from the samples had been isolated using a Total RNA kit (A&A Biotechnology, Poland) according to the manufacturer’s protocol. The isolated RNA samples then were dissolved in RNase-free water. The quantity of the isolated RNA was measured using NanoDrop (NanoDrop Technologies, USA). The samples with adequate amounts of RNA were treated with DNaseI to eliminate DNA contamination. The samples were subsequently purified with RNeasy MiniElute Cleanup Kit (Qiagen, Germany). During the final stage, the RNA samples were analyzed on a BioAnalyzer (Agilent, USA) to measure its quality and integrity. Only the samples of excellent quality were taken to the analysis (RIN, *RNA integration number* >9).

The total RNA (10 μg) of each tumour was reverse-transcribed using SuperScript Plus Direct cDNA Labeling System (Invitrogen, USA) according to the manufacturer’s protocol for each microarray slide. The control constituted the pooled RNA from each tumour (equal amounts). Single-strand cDNAs were stained with Alexa 647 and Alexa 555 (Invitrogen). Gene expression was assessed using UltraGAPS slides (Corning), spotted with a canine specific collection of 20,160 non-redundant clones of 3^′^-untranslated region (UTR) cDNA fragments [7].

Hybridization was performed with the automatic hybridization station HybArray12 (PerkinElmer, USA). Two replicates were made (dye-swap).

### Signal detection, quantification and analysis

The slides were analyzed with the use of microarray scanner ScanArray HT and ScanExpress software (PerkinElmer, USA).

For the purposes of unsupervised analysis (clustering) and the analysis of significantly regulated genes, background-corrected value of signal in each microarray channel was in use. Then, the log2 ratio of the sample versus control channels was calculated and the signal was normalized (loess normalization). The average log-ratio of the two dye-swap replicates was used as the signal for each of the patients. Prior to the analysis, non-specific filtering was performed, i.e. genes with small level of expression were removed (we set an arbitrary threshold according to which at least half of the samples’ log-ratios versus control was 2 or higher). Quality control, including MA analysis, and signal normalization were performed with the use of the Bioconductor software.

### Quantitative RT-PCR

The mRNA sequences of the key genes were obtained from NCBI database. Primers were designed using the PRIMER3 software (free online access), confirmed by Oligo Calculator (free online access) and Primer-Blast (NCBI database). Primers’ sequences are listed in Table 1. *Hprt* and *rps19* genes were used as non-regulated reference genes for normalization of target gene expression [8,9]. Quantitative RT-PCR was performed using fluorogenic Lightcycler Fast Strand DNA Syber Green (Roche) and the Light Cycler (Roche). The results were analyzed with the comparative Ct method [10]. Relative transcript abundance of the gene equals ΔCt values (ΔCt = Ct^reference^ – Ct^target^). Relative changes in transcript are expressed as ΔΔCt values (ΔΔCt = 2 ^-ΔCt^). The experiment was conducted three times.

**Table 1:** Primers used for real-time qPCR

**Gene symbol**	**Forward primer**	**Reverse primer**	**Optimum annealing temp. (°C)**	**Optimum annealing time (sec)**
*eif4b*	CTTTCTGGCTGAGGATGGAG	GGGCAGGTTCCCTAGAAAAG	59	10
*atf6*	GTCTCCAGCCTCCTCAAGTG	GGCTCTGCTAAGGAGGGACT	64	6
*tmem 85*	AATGGGACTGCTGCCTACAC	TCTGGGATTGGTCTCCTCAC	62	6
*hprt*	AGCTTGCTGGTGAAAAGGAC	TTATAGTCAAGGGCATATCC	59	6
*rps19*	CCTTCCTCAAAAAGTCTGGG	GTTCTCATCGTAGGGAGCAAG	61	10

Primers sequences used in this study and their annealing optimal temperature and time. The mRNA sequences of key genes were obtained from NCBI database. Primers were designed using PRIMER3 software (free online access) and checked using Oligo Calculator (free on-line access) and Primer-Blast (NCBI database). Primers sequences are listed in Table 1. *Hprt* and *rps19* genes were used as non-regulated reference genes for normalization of target gene expression [6,7].

### Statistical analysis

The statistical analysis of gene expression was performed using linear methods for microarrays (limma package in Bioconductor software) [11]. The mentioned method tests the null hypothesis of no differential expression between the groups of samples compared using the moderated t-statistic [11], which has similar interpretation as the ordinary *t*-test statistic. The expression of genes with the Benjamini-Hochberg (FDR) multiple-testing corrected p-value below 0.05 were qualified as significantly changed. The groups of samples compared in the analysis of differential expression were identified based on hierarchical clustering (unsupervised analysis) and were visually illustrated in the space of differentially expressed genes by a dendrogram.

The data discussed in this publication had been deposited in NCBI’s Gene Expression Omnibus and is freely accessible through GEO Series accession number GSE43778.

The gene function was identified using the NCBI database and PANTHER pathway analysis software [12]. The pathway analyses were conducted with the assistance of one-way ANOVA with the binominal Bonferroni statistic test (PANTHER) where the cut-off value p < 0.05 had been recognized.

The statistical analysis of Quantitative RT-PCR was conducted by use of the Prism version 5.00 software (GraphPad Software, USA). The one-way ANOVA, and ANOVA + Tukey HSD (Honestly Significant Difference) post-hoc test were applied, respectively. The p-value <0.05 was regarded as the significant data, whereas p-value <0.01 and p-value <0.001 as highly significant.

## Results

### Gene expression in canine mammary carcinomas of various grades of malignancy

**Figure 1:**
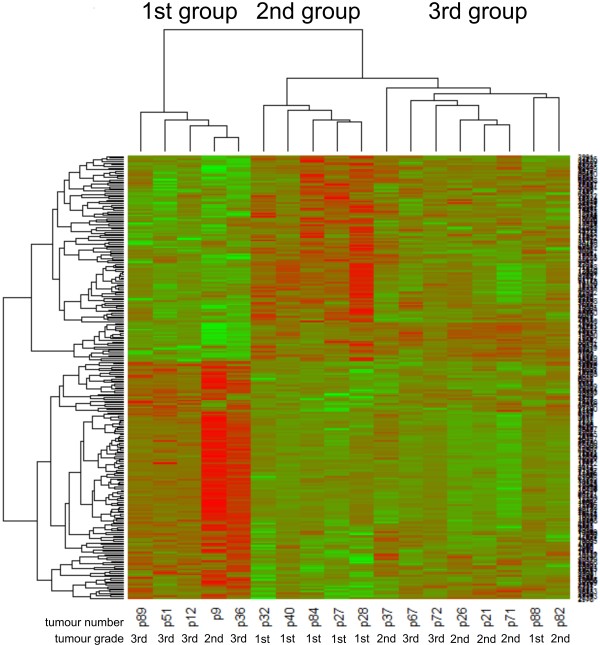
**Hierarchical analysis of expressed genes in canine mammary cancer of various grade of malignancy.** Variation in expression of genes in 18 experimental samples. Data are presented in a matrix format: each row represents a single gene, and each column an experimental sample. In each sample, the ratio of the abundance of transcripts of each gene to the median abundance of the gene’s transcript across all tissue samples, is represented by the colour of the corresponding cell in the matrix. Green squares, transcript levels below the median; red squares, transcript levels greater than the median. Colour saturation reflects the magnitude of the ratio relative to the median for each set of samples.

Hierarchical clustering (unsupervised analysis conducted in a blind manner) was performed on signal from all 18 patients and a gene tree dendrogram was generated (Figure 1). Based on the similarities between gene expression, three distinct clusters were generated: group 1 (tumour numbers: 9, 36, 89, 12 and 51); group 2 (tumour numbers: 32, 28, 27, 40 and 84) and group 3 (tumour numbers: 21, 71, 26, 37, 88, 72, 82, 67). Gene expression in these three groups have been compared against each other and the analysis (p < 0.01, *t*-test with Benjamini-Hochberg multiple testing correction) showed 160 differentially expressed genes between group 1 and 2 (85 up-regulated genes and 75 down-regulated genes; see Additional file 1: Tables S1 and S2), 372 differentially expressed genes between group 1 and 3 (232 up-regulated genes and 140 down-regulated genes; see Additional file 1: Tables S3 and S4) and 103 differentially expressed genes between group 2 and 3 (56 up-regulated and 47 down-regulated genes; see Additional file 1: Tables S5 and S6).

Comparison of the gene expression between all of these groups has pointed out n = 25 up-regulated genes in the group 1 compared with the other groups (2 and 3): ATP2C1, SIGLEC11, GPR155, RDH5, HERC4, ATF6, SIGLEC10, ARHGEF10L, ABHD7, SPLUNC3, HYOU1, ARHGAP15, NR6A1, GCSH, ZNF331, LSR, NR5A1, PLUNC, BNIPL, RFWD2, MYL6, MYL6B, GPI, SCNM1, XRCC2, MRPL40. The analysis exposed 8 down-regulated genes in the group 1 compared with the groups 2 and 3: TIMP2, HOXB7, MORN5, RIBC1, KSR2, HSDL2, ARL4C, SPTB.

### Comparison of molecular and pathological classifications of the tumours

Comparison of molecular and pathological classification highlighted that a tumour group 1 (gene expression cluster 1, Figure 1) was mainly consisted of grade 3 carcinomas (tumours no: 36, 89, 12 and 51) and one grade 2 carcinoma (tumour no. 9) (according to their morphological classification); tumour group 2 (cluster 2, Figure 1) included five grade 1 carcinomas (tumour numbers: 32, 28, 27, 40 and 84) (according to their morphological classification), whereas group 3 (cluster 3, Figure 1) was very heterogeneous and there were mainly grade 2 tumours (tumours no.: 21, 71, 26, 37 and 82), one grade 1 tumour (tumour no.: 88) and two grade 3 tumours (tumours no.: 72 and 67) (according to their morphological classification).

### Significant cellular pathways in which up/down-regulated genes are involved

PANTHER analysis showed cellular pathways in which significantly up/down-regulated genes were involved (Tables 2 and 3).

**Table 2:** Over-represented cellular pathways in canine mammary cancer

**Cellular pathway**	**1vs 2**	**1vs 3**	**2vs 3**
5HT1 type receptor mediated signaling pathway	X	X	
Acetate utilization		X	X
Alzheimer disease-amyloid secretase pathway		X	
Angiogenesis		X	X
Apoptosis signaling pathway	X	X	X
Axon guidance mediated by semaphorins		X	X
Beta1 adrenergic receptor signaling pathway	X	X	
Beta2 adrenergic receptor signaling pathway	X	X	
Cadherin signaling pathway		X	X
Cytoskeletal regulation by Rho GTPase		X	X
DNA replication		X	X
Endothelin signaling pathway		X	X
GABA-B_receptor_II_signaling	X	X	
Glycolysis	X	X	X
Heterotrimeric G-protein signaling pathway-Gi alpha and Gs alpha mediated pathway	X	X	X
Heterotrimeric G-protein signaling pathway-Gq alpha and Go alpha mediated pathway	X	X	
Histamine H2 receptor mediated signaling pathway	X	X	
Huntington disease		X	X
Inflammation mediated by chemokine and cytokine signaling pathway	X	X	X
Interleukin signaling pathway		X	X
Metabotropic glutamate receptor group II pathway	X	X	
Metabotropic glutamate receptor group III pathway	X	X	
Muscarinic acetylcholine receptor 2 and 4 signaling pathway	X	X	
P53 pathway feedback loops 1	X	X	
PDGF signaling pathway	X	X	
PI3 kinase pathway	X	X	X
Pentose phosphate pathway	X	X	
Ras Pathway	X	X	
Transcription regulation by bZIP transcription factor		X	X
VEGF signaling pathway		X	X
Wnt signaling pathway	X	X	X
p53 pathway feedback loops 2		X	X
p53 pathway		X	X

Cellular pathways in which are involved significantly up-regulated genes in the examined tumour groups. The analysis was conducted using PANTHER Database (http://www.pantherdb.org). The pathway activity in the tumour group 1 vs 2, 1 vs 3 and 2 vs 3 is marked as X.

**Table 3:** Under-represented cellular pathways in canine mammary cancer

**Cellular pathways**	**1vs 2**	**1vs 3**	**2vs 3**
5HT1 type receptor mediated signaling pathway		X	X
Alzheimer disease-amyloid secretase pathway	X	X	
Alzheimer disease-presenilin pathway	X	X	
Angiogenesis	X	X	
Apoptosis signaling pathway	X	X	
B cell activation	X	X	
Beta1 adrenergic receptor signaling pathway		X	X
Beta2 adrenergic receptor signaling pathway		X	X
Cadherin signaling pathway	X	X	
EGF receptor signaling pathway	X	X	
Endothelin signaling pathway	X	X	X
GABA-B_receptor_II_signaling		X	X
Glycolysis	X	X	
Hedgehog signaling pathway		X	X
Heterotrimeric G-protein signaling pathway-Gi alpha and Gs alpha mediated pathway		X	X
Heterotrimeric G-protein signaling pathway-Gq alpha and Go alpha mediated pathway	X		X
Histamine H2 receptor mediated signaling pathway		X	X
Huntington disease	X	X	
Hypoxia response via HIF activation		X	X
Inflammation mediated by chemokine and cytokine signaling pathway	X	X	
Metabotropic glutamate receptor group II pathway		X	X
Metabotropic glutamate receptor group III pathway		X	X
Muscarinic acetylcholine receptor 2 and 4 signaling pathway		X	X
Oxidative stress response	X	X	X
PDGF signaling pathway	X	X	
Parkinson disease	X	X	
T cell activation	X	X	
Thyrotropin-releasing hormone receptor signaling pathway	X		
Transcription regulation by bZIP transcription factor		X	X
Wnt signaling pathway	X	X	
p53 pathway by glucose deprivation	X	X	

Cellular pathways in which are involved significantly down-regulated genes in the examined tumour groups. The analysis was conducted using PANTHER Database (http://www.pantherdb.org). The pathway activity in the tumour group 1 vs 2, 1 vs 3 and 2 vs 3 is marked as X.

The up-regulated genes in tumour group 1 were involved mainly in following pathways: 5HT1 receptor, apoptosis, β1 and β2 adrenergic receptors, G protein, GABA-B receptor, H2 histamine receptor, metabotropic II and III receptor, acetylocholine M2 and M4 receptor, P53, PDGF and RAS (Table 2).

The down-regulated genes in group 1 were mainly involved in following pathways: Alzheimer (amyloid and presenilin), angiogenesis, apoptosis, B lymphocyte activation, E-cadherin, EGF receptor, Huntington, inflammation-mediated, PDGF, Parkinson, T lymphocytes activation, Wnt, P53 and glucose-deprivation-mediated (Table 3).

### Quantitative RT-PCR

**Figure 2:**
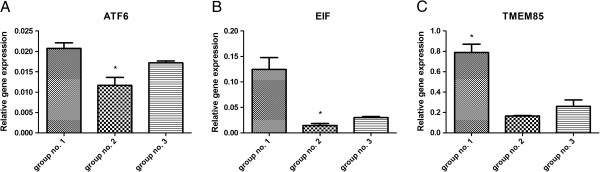
**Expression of selected genes assessed using real-time qPCR.** Expression of randomly selected genes in canine mammary carcinoma of various grade of malignancy. The changes in gene expression differed significantly (p < 0.05, *N =* 3).

Quantitative RT-PCR analysis confirmed similar trends in the expression of randomly selected genes: ATF6, EIF and TMEM85 (Figure 2).

The highest relative expression of ATF6 showed tumours classified into the group 1 (0.210 ± 0.0014), whereas the lowest expression showed tumours classified into the group 2 (0.012 ± 0.0014). The relative mean expression of ATF6 in tumours classified into the group 3 was 0.020 (±0.0005). The relative expression of ATF6 differed significantly between tumours classified into the groups 1 and 2 (p < 0.05).

The highest expression of EIF showed tumours classified into the group 1 (0.125 ± 0.0230), whereas the lowest expression showed tumours classified into the group 2 (0.015 ± 0.0040). Mean relative expression of EIF in tumours classified into the group 3 was 0.030 (±0.0020). The mean relative expression of EIF differed significantly between tumours classified into the groups 1 and 2 as well as classified into the groups 1 and 3 (p < 0.05).

The highest expression of TMEM85 showed tumours classified into the group 1 (0.790 ± 0.0800), whereas the lowest showed tumours classified into the group 2 (0.170 ± 0.0100). Mean relative expression of EIF in tumours classified into the group 3 was 0.260 (±0.0600). The mean relative expression of EIF differed significantly between tumours classified into the groups 1 and 2 as well as between tumours classified into the groups 1 and 3 (p < 0.05).

## Discussion

Canine mammary cancer constitute more than 40% of all malignancies in dogs [13] thus this is a serious clinical problem. Unfortunately, its molecular biology has not been fully recognized yet. So far, there are only three reports of wide gene expression analysis in canine mammary tumour tissues [14-16]. The most interesting study was conducted by Klopfleisch et al. [15]. The authors identified a gene expression profile of canine mammary tumours which was associated with an early metastatic spread to the lymph nodes. This “expression profile” can be used as a marker of metastasis prediction. In the opposite reflection, as far as we are aware, there have not been published any results regarding comparison between pathological and molecular diagnosis in canine mammary malignancy.

Thus, the objectives of our study were three-fold: 1) to assess gene expression profiles of canine mammary carcinomas of various grade of malignancy, 2) to perform unsupervised hierarchical classification based on their molecular portraits, and 3) to compare their molecular classification with the pathological diagnosis.

**Figure 3:**
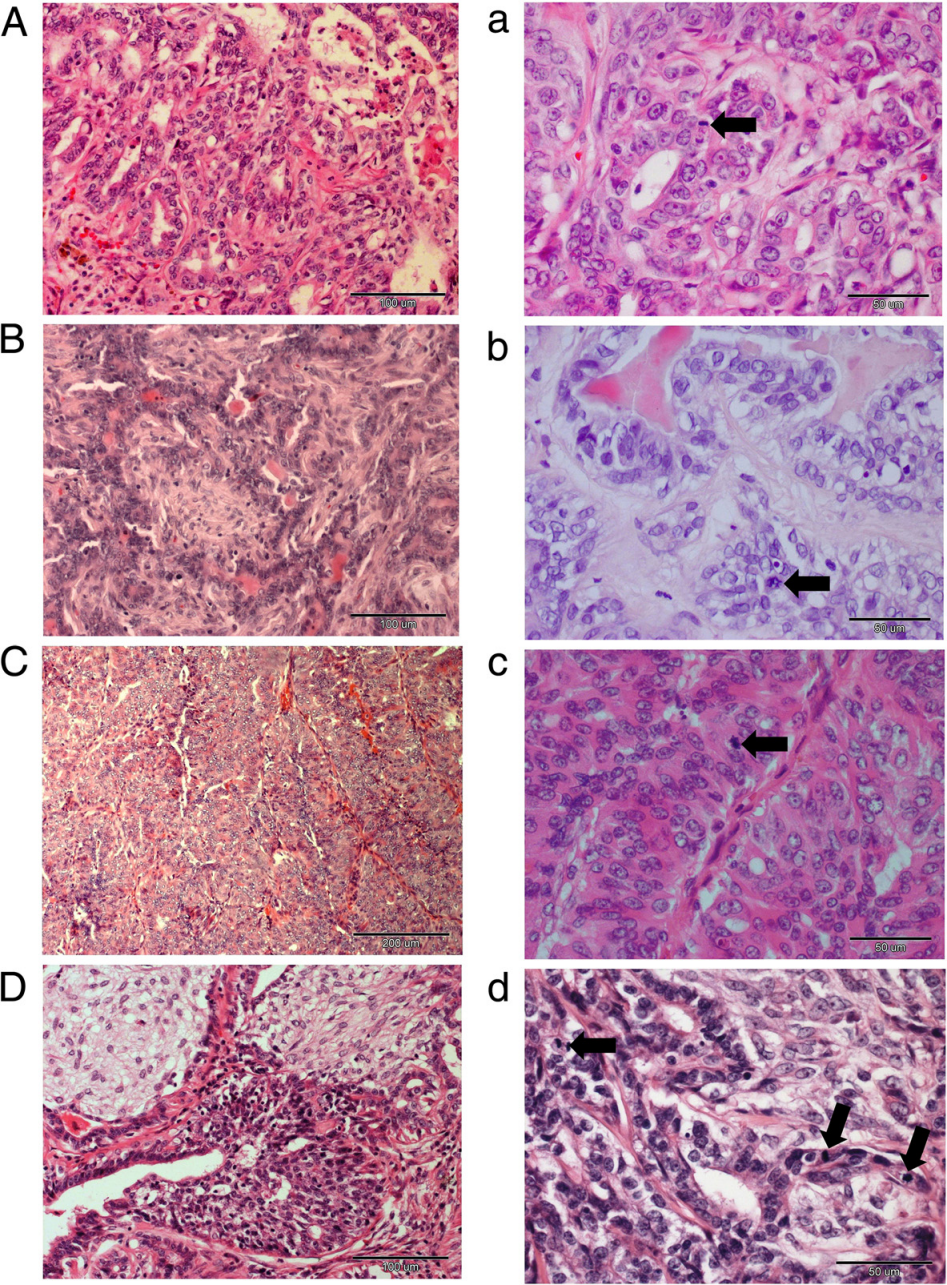
**The canine mammary carcinoma tissues which pathological diagnosis differed from molecular classification.** The pictures of canine mammary carcinoma tissues (haematoxylin-eosin staining) which pathological diagnosis differed from their molecular classification. **A.** Tumour no 9. (pathologically grade 2 complex carcinoma, classified to the most malignant group). Moderate tubule formation was observed (×200). **a)** Neoplastic cells exhibited moderate nuclear pleomorphism, mild to moderate hyperchromasia with noticeable nucleoli was observed. Eight mitoses per 10 HPF were present. Mitotic cell is indicated by the arrow (400×). **B.** Tumour no. 88 (pathologically grade 1 complex carcinoma, classified to the third group). Moderate tubule formation was observed. Epithelial cells were arranged in irregular tubules lined by a single to few layers of cells. Some tubules contained an eosinophilic secretion (200×). **b)** Neoplastic epithelial cells had regular small nuclei (round to ovoid) with small or indistinct nucleoli. Presence of 1 mitose per 10 HPF. Mitotic cell was indicated by the arrow (400×). **C.** Tumour no. 72: pathologically grade 3 simple carcinoma, classified to the third group. A few tubules were observed. In some areas neoplastic cells were closely packed and arranged in solid foci (100×). **c)** Marked nuclear pleomorphism was observed as well as cells containing stippled chromatin and variably distinct nucleoli. Twenty five mitoses per 10 HPF were counted (arrows). (400×). **D.** Tumour no. 67: pathologically grade 3 complex carcinoma, classified to the third group. Moderate tubule formation was observed (200×). **d)** Marked nuclear pleomorphism and presence of nuclei with hyperchromasia were observed. Twenty eight mitoses per 10 HPF were noted (arrows) (400×).

The results of our unsupervised analysis allowed the classification of the examined tissues into three groups. The first one (tumour group no. 1) significantly differed from the others and consisted of four grade 3 carcinomas and one grade 2 carcinoma (these tumours showed classical pathological features of higher biological aggressiveness). The second group (tumour group no. 2) consisted of four grade 1 carcinomas. The last one (tumour group no. 3) was very heterogeneous (based on the pathological diagnosis of these tumours) and consisted of two grade 1 carcinomas, two grade 3 carcinomas and five grade 2 carcinomas. Hierarchical clustering presented the view that the gene expression in the tumour groups no. 2 and no. 3 was relatively similar, whereas gene expression in the tumour group no. 1 (the tumour group showing classical pathological features of higher biological aggressiveness) differed significantly (Figure 1; Additional file 1: Tables S1–S6). Our results showed that pathologically different tumours may have similar gene expression and vice versa (Figure 1, Figure 3) what can be particularly observed in tumour group no. 3. These results can explain why sometimes very similar (pathologically) tumours respond for treatment in a different manner. We also consider these results as partially related with the fact that the tissue sample taken to the pathological examination is not the same that was taken to the microarray analysis. However, neighbour tissue samples were collected for both analyses. Moreover, within the four differently diagnosed tumours only one sample was of a mixed nature (tumour no. 88). Thus, in our opinion the risk of probes contamination in the other three cases is unlikely.

For clinicians, the most important seems to be the tumour group no. 1, as these tumours have shown classical pathological features of higher biological aggressiveness. The gene expression of these tumours also differed significantly from the other groups, thus only these data files are being as the discussion in this documentation sheet. From our perspective a few of up-regulated genes seem to be particularly interesting.

Among 25 up-regulated genes in tumours classified into group no. 1, compared to the other two groups, PANTHER software recognized two of them as responsible for chemotherapy resistance (ATP2C1 and ABHD7), two associated with myeloid cells infiltration of tumour mass (SIGLEC10 and SIGLEC11), one related to hypoxia (HYOU1), four genes related to cancer cell motility (MYL6, MYL6B, GPI and ARHGAP), one associated with DNA-repair (XRCC2), one with p53-signalling (RFWD2) and three transcriptional factors (NR6A1, RDH5, CYP19).

As of this matter what we do believe in, is the fact that important up-regulated genes in tumour group no. 1 were: SIGLEC10 and SIGLEC11. These sialic acid-binding Immunoglobulin-like lectines are expressed on the myeloid-origin cell surface [17]. All of the identified SIGLEC proteins are very similar to CD33 protein, which is a marker of myeloid cells. For example, SIGLEC10 expression was found in eosinophils and B lymphocytes, whereas expression of SIGLEC11 was found mainly in tissue macrophages. Expression of these proteins in cancer may be related with increased recruitment of myeloid cells into the tumour mass. The tumour is composed of various cells depending on the tumour type, but myeloid cells form a major component [18]. Clinical studies have shown a correlation between the number of myeloid cells (mainly macrophages) and poor prognosis in following cancers: breast, prostate, ovarian, cervical, endometrial, oesophageal, urinary bladder [19-23]. Our previous studies had pointed out the significantly higher expression level of myeloid-specific antigens in grade 3 canine mammary tumours [16,24]. It indicates that in dogs the number of tumour-infiltrating myeloid cells or expression of myeloid-specific antigens by cancer cells may constitute a new marker of malignancy. Based on the previous outcomes it can be suggested that cancer cells may acquire phenotype of myeloid cells which, as the matter of fact, was demonstrated by expression of myeloid cell antigens on their surface [25]. One of the explanation why cancer cells may exhibit myeloid cell-specific antigens is that these cells fuse, forming hybrids that adopt phenotypic features of both parental cells [26-28]. The tumour cells that express myeloid antigens may also exhibit other phenotypic characteristics of myeloid cells, such as capabilities of cell rolling, spreading, dissociation, diapedesis, migration and matrix invasion [25].

The most malignant tumours are usually more resistant to anticancer therapy [29]. Thus, from our perspective two up-regulated genes in the tumour group no. 1 may be important: ATP2C1 and ABHD7. In breast cancer ATP2C1 ensures resistance to paclitaxel [30]. A product of the other gene (ABHD7) is responsible for cellular detoxication after drug administration (mainly compounds containing aromatic rings) [31]. Obviously, in cancer cell it can be responsible for the metabolism of anti-cancer drugs (e.g. anthracyclines which contains aromatic rings), decreasing their activity [31]. These results correlate with a clinical picture of canine mammary malignant tumours [32].

Interestingly, in the tumour group no. 1 we highlighted a significant over-manifestation of genes involved in the β1 and β2 adrenergic receptors signalling, H2 histamine receptor signalling, and acetylcholine M2 and M4 receptor signalling. The possible important role of neurotransmitters in cancer progression and metastasis had been previously described [33,34]. Neurotransmitters are regulators of cell migration in a similar manner as chemokines. Unfortunately, only a few reports are available on the neurotransmitter signalling in tumour tissues. Our previous study [34] had indicated that carcinoma-associated fibroblasts increased the expression of neurotransmitters in cancer cells. A role of stress hormones in carcinogenesis and tumour progression is commonly known. It has been shown that norepinephrine strongly induced migration of tumour cells [35,36], whereas epinephrine modulated carcinogenesis in the lungs [37]. Thus, stress is considered as a major risk factor of cancer development [34].

## Conclusions

Molecular classification of canine mammary tumours is not identical with pathological classification. The tumours classified to the group no. 1 (showing pathological parameters classically associated to high biological aggressiveness) constituted a molecularly homogeneous group which differs significantly from the others. The most important up-regulated genes in these tumours were mainly involved in myeloid cell phenotype, neurotransmitters pathways as well as in anti-cancer drug resistance.

## Competing interests

The authors declare that they have no competing interests.

## Authors’ contributions

KP: research design, RNA isolation, microarrays analysis, real-time qPCR analysis, primers design, data analysis, manuscript preparation; HM: statistical analysis of microarray experiment; AM: microarrays analysis; ID: pathological analysis of the tumour samples; JM: microarrays printing; TM: research design; MK: research design, statistical analysis, manuscript preparation. All authors read and approved the final manuscript.

## Supplementary Material

Additional file 1: Table S1 The list and characteristics of genes up-regulated in tumours group no. 1 in comparison to tumours group no. 2. **Table S2.** The list and characteristics of genes down-regulated in tumours group no. 1 in comparison to tumours group no. 2. **Table S3.** The list and characteristics of genes up-regulated in tumours group no. 1 in comparison to tumours group no. 3. **Table S4.** The list and characteristics of genes down-regulated in tumours group no. 1 in comparison to tumours group no. 3. **Table S5.** The list and characteristics of genes up-regulated in tumours group no. 2 in comparison to tumours group no. 3. **Table S6.** The list and characteristics of genes down-regulated in tumours group no. 2 in comparison to tumours group no. 3. Click here for file
