# Supplementary material for: Gene expression profiles in canine mammary carcinomas of various grades of malignancy
Source: BMC Vet Res. 2013 Apr 15;9:78. doi: 10.1186/1746-6148-9-78 (PMC3691656; doi:10.1186/1746-6148-9-78)
Supplement: Additional file 1: Table S1 — The list and characteristics of genes up-regulated in tumours group no. 1 in comparison to tumours group no. 2. Table S2. The list and characteristics of genes down-regulated in tumours group no. 1 in comparison to tumours group no. 2. Table S3. The list and characteristics of genes up-regulated in tumours group no. 1 in comparison to tumours group no. 3. Table S4. The list and characteristics of genes down-regulated in tumours group no. 1 in comparison to tumours group no. 3. Table S5. The list and characteristics of genes up-regulated in tumours group no. 2 in comparison to tumours group no. 3. Table S6. The list and characteristics of genes down-regulated in tumours group no. 2 in comparison to tumours group no. 3. [file 1746-6148-9-78-S1.doc]

# Supplementary Tables

## Table S1 - The list and characteristics of genes up-regulated in tumours group no. 1 in comparison to tumours group no. 2.

Symbol, name and characteristics of up-regulated genes in group no. 1 in comparison to tumour group no. 2. The biological processes and molecular functions of the genes are based on the PANTHER database (www.pantherdb.org). Only significantly regulated genes were included in this table (p<0.01). The genes significantly up-regulated in comparison to the tumour group no. 3 are bolded.

| **Gene symbol** | **Gene name** | **Molecular function** | **Biological process** |
| --- | --- | --- | --- |
| DPP6 | Dipeptidyl aminopeptidase-like protein 6 | peptidase activity;  protein binding | immune system process; neurological system process;cation transport; cell surface receptor linked signal transduction; protein metabolic process; signal transduction; cellular defense response |
| **ATP2C1** | Calcium-transporting ATPase type 2C member 1 | hydrolase activity; cation transmembrane transporter activity; ion channel activity | cation transport; lipid transport; lipid metabolic process; cellular calcium ion homeostasis |
| **SIGLEC11** | Sialic acid-binding Ig-like lectin 11 | receptor activity; structural constituent of myelin sheath; receptor binding | B cell mediated immunity; cell surface receptor linked signal transduction; cell-cell adhesion; signal transduction; cell-cell adhesion; response to stimulus |
| **GPR155** | Integral membrane protein GPR155 | receptor activity | unclear biological function |
| TM2D3 | TM2 domain-containing protein 3 | unclear molecular function | neurological system process; induction of apoptosis |
| **RDH5** | 11-cis retinol dehydrogenase | oxidoreductase activity | visual perception; sensory perception; lipid metabolic process |
| ARHGEF6 | Rho guanine nucleotide exchange factor 6 | receptor binding; small GTPase regulator activity; guanyl-nucleotide exchange factor activity | B cell mediated immunity; neurological system process; cell surface receptor linked signal transduction; intracellular signaling cascade; cell motion; signal transduction; cellular defense response |
| AP1S2 | AP-1 complex subunit sigma-2 | unclear molecular function | intracellular protein transport; endocytosis |
| **HERC4** | Probable E3 ubiquitin-protein ligase HERC4 | ubiquitin-protein ligase activity | protein metabolic process; ectoderm development; mesoderm development; skeletal system development; nervous system development |
| ATP5J | ATP synthase-coupling factor 6, mitochondrial | catalytic activity; cation transmembrane transporter activity; hydrogen ion transmembrane transporter activity | oxidative phosphorylation; respiratory electron transport chain; cation transport |
| CEP70 | Centrosomal protein of 70 kDa | unclear molecular function | unclear biological function |
| TRIM44 | Tripartite motif-containing protein 44 | ubiquitin-protein ligase activity; structural constituent of cytoskeleton; nucleic acid binding; cytoskeletal protein binding | neurotransmitter secretion; intracellular protein transport; exocytosis; cell cycle; signal transduction; synaptic transmission; carbohydrate metabolic process; protein metabolic process; cell cycle; signal transduction; cell-cell signaling; dorsal/ventral axis specification; mesoderm development; mammary gland development |
| **ATF6** | Processed cyclic AMP-dependent transcription factor ATF-6 alpha | DNA binding; transcription factor activity | immune system process; neurological system process; signal transduction; nucleobase, nucleoside, nucleotide and nucleic acid metabolic process; signal transduction; ectoderm development; nervous system development; response to stress |
| SLMAP | Sarcolemmal membrane-associated protein | unclear molecular function | unclear biological function |
| NFKBID | NF-kappa-B inhibitor delta | DNA binding; protein binding; transcription factor activity | protein metabolic process |
| **SIGLEC10** | Sialic acid-binding Ig-like lectin 10 | receptor activity; structural constituent of myelin sheath; receptor binding | B cell mediated immunity; cell surface receptor linked signal transduction; cell-cell adhesion; signal transduction; cell-cell adhesion; response to stimulus |
| STAU1 | Double-stranded RNA-binding protein Staufen homolog 1 | hydrolase activity; deaminase activity; DNA binding; RNA binding; protein binding; kinase activator activity; kinase regulator activity | spermatogenesis; response to interferon-gamma; neurological system process; apoptosis; cell cycle; nucleobase, nucleoside, nucleotide and nucleic acid metabolic process; protein metabolic process; cell cycle; anterior/posterior axis specification; response to stimulus; RNA localization |
| MRPS31 | 28S ribosomal protein S31, mitochondrial | structural constituent of ribosome; nucleic acid binding | protein metabolic process |
| ARHGEF10L | Rho guanine nucleotide exchange factor 10-like protein | peptidase activity | protein metabolic process |
| CPA5 | Carboxypeptidase A5 | peptidase activity | protein metabolic process |
| GNB1 | Guanine nucleotide-binding protein G(I)/G(S)/G(T) subunit beta-1 | GTPase activity; protein binding | sensory perception; cell surface receptor linked signal transduction; signal transduction |
| RPL10A | 60S ribosomal protein L10a | structural constituent of ribosome; nucleic acid binding | nucleobase, nucleoside, nucleotide and nucleic acid metabolic process; protein metabolic process |
| SLC25A23 | Calcium-binding mitochondrial carrier protein SCaMC-3 | amino acid transmembrane transporter activity; transmembrane transporter activity | cation transport; phosphate transport; lipid transport; nucleobase, nucleoside, nucleotide and nucleic acid transport; phosphate metabolic process; nucleobase, nucleoside, nucleotide and nucleic acid metabolic process; lipid metabolic process |
| MLLT1 | Protein ENL | DNA binding; chromatin binding; transcription factor activity | nucleobase, nucleoside, nucleotide and nucleic acid metabolic process |
| **ABHD7** | Abhydrolase domain-containing protein 7 | peptidase activity | immune system process; protein metabolic process; response to toxin |
| EIF | Membrane-associated guanylate kinase, WW and PDZ domain-containing protein 3 | kinase activity | neurological system process; intracellular signaling cascade; synaptic transmission; signal transduction; cell-cell signaling |
| EXOSC4 | Exosome complex exonuclease RRP41 | nucleotidyltransferase activity; hydrolase activity,  acting on ester bonds; nucleic acid binding | nucleobase, nucleoside, nucleotide and nucleic acid metabolic process |
| **SPLUNC3** | Short palate, lung and nasal epithelium carcinoma-associated protein 3 | carbohydrate transmembrane transporter activity; transmembrane transporter activity | macrophage activation; lipid transport; lipid metabolic process; response to stress; defense response to bacterium |
| SLC12A6 | Solute carrier family 12 member 6 | cation transmembrane transporter activity | cation transport |
| TMEM85 | Transmembrane protein 85 | unclear molecular function | unclear biological process |
| ISYNA1 | Inositol-3-phosphate synthase 1 | isomerase activity | lipid metabolic process |
| ZNF248 | Zinc finger protein 248 | DNA binding; transcription factor activity | spermatogenesis;nucleobase, nucleoside, nucleotide and nucleic acid metabolic process |
| **HYOU1** | Hypoxia up-regulated protein 1 | unclear molecular function | immune system process; protein metabolic process; response to stress |
| CORO7 | Coronin-7 | structural constituent of cytoskeleton; cytoskeletal protein binding | intracellular protein transport; endocytosis; mitosis; signal transduction; cell motion; mitosis; signal transduction; cellular component morphogenesis;  cellular component morphogenesis;  cellular component morphogenesis |
| CPA4 | Carboxypeptidase A4 | peptidase activity | protein metabolic process |
| CPA1 | Carboxypeptidase A1 | peptidase activity | protein metabolic process |
| **ARHGAP15** | Rho GTPase-activating protein 15 | protein binding;small GTPase regulator activity | intracellular signaling cascade; signal transduction |
| **NR6A1** | Nuclear receptor subfamily 6 group A member 1 | ligand-dependent nuclear receptor activity; DNA binding; transcription factor activity | spermatogenesis; intracellular signaling cascade;  nucleobase, nucleoside, nucleotide and nucleic acid metabolic process;  signal transduction |
| **GCSH** | Glycine cleavage system H protein, mitochondrial | hydrolase activity | cellular amino acid and derivative metabolic process |
| CUTC | Copper homeostasis protein cutC homolog | cation transmembrane transporter activity | cation transport |
| **ZNF331** | Zinc finger protein 331 | DNA binding; transcription factor activity | nucleobase, nucleoside, nucleotide and nucleic acid metabolic process |
| BTBD10 | BTB/POZ domain-containing protein 10 | unclear molecular function | protein metabolic process |
| AFG3L2 | AFG3-like protein 2 | peptidase activity | intracellular protein transport; protein metabolic process;  organelle organization; mitochondrion organization |
| CNOT4 | CCR4-NOT transcription complex subunit 4 | DNA binding; transcription factor activity | nucleobase, nucleoside, nucleotide and nucleic acid metabolic process |
| LSR | Lipolysis-stimulated lipoprotein receptor | unclear molecular function | unclear biological function. |
| HLA-DRB5 | HLA class II histocompatibility antigen, DRB5 β chain | unclear molecular function | antigen processing and presentation of peptide or polysaccharide antigen via MHC class II; cellular defense response |
| CISD2 | CDGSH iron sulfur domain-containing protein 2 | unclear molecular function | unclear biological function |
| ZNF25 | Zinc finger protein 25 | DNA binding; transcription factor activity | spermatogenesis; nucleobase, nucleoside, nucleotide and nucleic acid metabolic process |
| ZNF33A | Zinc finger protein 33A | DNA binding; transcription factor activity | nucleobase, nucleoside, nucleotide and nucleic acid metabolic process |
| **NR5A1** | Steroidogenic factor 1 | ligand-dependent nuclear receptor activity; DNA binding; transcription factor activity | signal transduction;  nucleobase, nucleoside, nucleotide and nucleic acid metabolic process |
| MAP1A | MAP1 light chain LC2 | structural constituent of cytoskeleton; cytoskeletal protein binding | cellular component morphogenesis |
| **PLUNC** | Protein Plunc | carbohydrate transmembrane transporter activity; transmembrane transporter activity | macrophage activation; lipid transport; lipid metabolic process; response to stress; defense response to bacterium |
| **BNIPL** | Bcl-2/adenovirus E1B 19 kDa-interacting protein 2-like protein | unclear molecular function | apoptosis |
| WDR23 | WD repeat-containing protein 23 | structural molecule activity | unclear biological function |
| CTBP2 | C-terminal-binding protein 2 | oxidoreductase activity;  DNA binding;  transcription factor activity;  transcription cofactor activity | carbohydrate metabolic process; cellular amino acid and derivative metabolic process |
| SAPS1 | Serine/threonine-protein phosphatase 6 regulatory subunit 1 | protein binding; phosphatase regulator activity | unclear biological function. |
| CTH | Cystathionine gamma-lyase | lyase activity | cellular amino acid and derivative metabolic process |
| **RFWD2** | E3 ubiquitin-protein ligase RFWD2 | hydrolase activity, acting on ester bonds;  ubiquitin-protein ligase activity; RNA splicing factor activity, transesterification mechanism; RNA binding; protein binding; kinase inhibitor activity; kinase regulator activity | nucleobase, nucleoside, nucleotide and nucleic acid metabolic process; protein metabolic process; ectoderm development; nervous system development; RNA localization |
| KCNB1 | Potassium voltage-gated channel subfamily B member 1 | cation transmembrane transporter activity; voltage-gated potassium channel activity; cation channel activity | muscle contraction; blood circulation; neuronal action potential propagation; cation transport;signal transduction; synaptic transmission; signal transduction; cell-cell signaling |
| **MYL6** | Myosin light polypeptide 6 | structural constituent of cytoskeleton; calcium ion binding; calmodulin binding | muscle contraction |
| **MYL6B** | Myosin light chain 6B | structural constituent of cytoskeleton; calcium ion binding; calmodulin binding | muscle contraction |
| BDH2 | 3-hydroxybutyrate dehydrogenase type 2 | oxidoreductase activity | metabolic process |
| CES7 | Carboxylesterase 7 | hydrolase activity, acting on ester bonds; receptor binding | immune system process; neurological system process; meiosis; signal transduction; synaptic transmission; cell adhesion; vitamin metabolic process; lipid metabolic process; meiosis; signal transduction; cell-cell signaling; cell adhesion; ectoderm development; nervous system development; response to toxin |
| BUB1 | Mitotic checkpoint serine/threonine-protein kinase BUB1 | kinase activity | mitosis; protein metabolic process; mitosis; chromosome segregation; embryonic development |
| **GPI** | Glucose-6-phosphate isomerase | isomerase activity | carbohydrate metabolic process |
| **SCNM1** | Sodium channel modifier 1 | unclear molecular function | unclear biological process |
| RNF41 | E3 ubiquitin-protein ligase NRDP1 | unclear molecular function | unclear biological function |
| **XRCC2** | DNA repair protein XRCC2 | hydrolase activity; DNA binding | immune system process; meiosis; nucleobase, nucleoside, nucleotide and nucleic acid metabolic process; meiosis; response to stress |
| ANKRD10 | Ankyrin repeat domain-containing protein 10 | unclear molecular function | unclear biological function |
| **MRPL40** | 39S ribosomal protein L40, mitochondrial | structural constituent of ribosome; nucleic acid binding | protein metabolic process |
| PLEKHA2 | Pleckstrin homology domain-containing family A member 2 | unclear molecular function | unclear biological function |
| EXOC2 | Exocyst complex component 2 | unclear molecular function | intracellular protein transport; exocytosis |
| TM6SF1 | Transmembrane 6 superfamily member 1 | unclear molecular function | unclear biological function |
| UBL4B | Ubiquitin-like protein 4B | structural constituent of ribosome; nucleic acid binding | protein metabolic process |
| CPA2 | Carboxypeptidase A2 | peptidase activity | protein metabolic process |

## Table S2 - The list and characteristics of genes down-regulated in tumours group no. 1 in comparison to tumours group no. 2.

Symbol, name and characteristics of down-regulated genes in tumour group no. 1 in comparison to tumour group no. 2. The biological processes and molecular functions of the genes are based on the PANTHER database (www.pantherdb.org). Only significantly regulated genes were included in this table (p<0.01).

| **Gene symbol** | **Gene name** | **Molecular function** | **Biological process** |
| --- | --- | --- | --- |
| CRYAB | Alpha-crystallin B chain | structural molecule activity | immune system process; muscle contraction; visual perception; sensory perception; protein metabolic process; response to stress |
| ARPP21 | cAMP-regulated phosphoprotein 21 | unclear molecular function | nucleobase, nucleoside, nucleotide and nucleic acid metabolic process |
| CDH1 | E-Cad/CTF3 | G-protein coupled receptor activity; calcium ion binding | visual perception; sensory perception of sound; sensory perception; cell surface receptor linked signal transduction; cell-cell adhesion; cell motion; signal transduction; cell-cell adhesion; cellular component morphogenesis; cellular component morphogenesis; ectoderm development; mesoderm development; cellular component morphogenesis; embryonic development; nervous system development; heart development; muscle organ development |
| SSPN | Sarcospan | unclear molecular function | unclear biological function |
| ADD2 | Beta-adducin | structural constituent of cytoskeleton; cytoskeletal protein binding | protein metabolic process |
| TIMP2 | Metalloproteinase inhibitor 2 | protein binding; peptidase inhibitor activity | protein metabolic process |
| DYNC1I2 | Cytoplasmic dynein 1 intermediate chain 2 | structural constituent of cytoskeleton | intracellular protein transport; vesicle-mediated transport; cell cycle; nucleobase, nucleoside, nucleotide and nucleic acid metabolic process; cell cycle; cellular component morphogenesis; RNA localization |
| GALNT11 | Polypeptide N-acetylgalactosaminyltransferase 11 | transferase activity, transferring glycosyl groups | carbohydrate metabolic process; protein metabolic process |
| RPL12 | 60S ribosomal protein L12 | structural constituent of ribosome; nucleic acid binding | protein metabolic process |
| DCTN4 | Dynactin subunit 4 | structural constituent of cytoskeleton; cytoskeletal protein binding | intracellular protein transport; vesicle-mediated transport; cellular component morphogenesis |
| ACTG2 | Actin, gamma-enteric smooth muscle | structural constituent of cytoskeleton | intracellular protein transport; exocytosis; endocytosis; mitosis; cytokinesis; mitosis; cellular component morphogenesis |
| HOXB7 | Homeobox protein Hox-B7 | DNA binding; transcription factor activity | female gamete generation; nucleobase, nucleoside, nucleotide and nucleic acid metabolic process; segment specification; ectoderm development; gut mesoderm development; embryonic development; skeletal system development; angiogenesis; nervous system development; muscle organ development |
| NEURL2 | Neuralized-like protein 2 | ubiquitin-protein ligase activity | gamete generation; cell motion; ectoderm development; nervous system development |
| YWHAQ | 14-3-3 protein theta | Molecular function unclissified | cell cycle; signal transduction; cell cycle; signal transduction |
| GABARAP | Gamma-aminobutyric acid receptor-associated protein | structural constituent of cytoskeleton; cytoskeletal protein binding | unclear biological functiond |
| KIAA1239 | Leucine-rich repeat and WD repeat-containing protein KIAA1239 | unclear molecular function | unclear biological function |
| MADD | MAP kinase-activating death domain protein | protein binding; small GTPase regulator activity; guanyl-nucleotide exchange factor activity | neurological system process; induction of apoptosis; intracellular signaling cascade; synaptic transmission; signal transduction; cell-cell signaling; ectoderm development; nervous system development |
| SPINK6 | Serine protease inhibitor Kazal-type 6 | protein binding | unclear biological function |
| MORN5 | MORN repeat-containing protein 5 | kinase activity | cell surface receptor linked signal transduction; signal transduction |
| ERLIN2 | Erlin-2 | unclear molecular function | unclear biological function |
| EGFLAM | Pikachurin | unclear molecular function | unclear biological function |
| NDUFV2 | NADH dehydrogenase [ubiquinone] flavoprotein 2, mitochondrial | oxidoreductase activity | oxidative phosphorylation; respiratory electron transport chain |
| DAG1 | Beta-dystroglycan | receptor activity | signal transduction; cell-matrix adhesion; signal transduction; cell-matrix adhesion |
| S100A4 | Protein S100-A4 | calcium ion binding; receptor binding; calmodulin binding | immune response; macrophage activation; cell cycle; intracellular signaling cascade; cell motion; cell cycle; signal transduction; response to stimulus |
| KBTBD10 | Kelch repeat and BTB domain-containing protein 10 | peptidase activity; DNA topoisomerase activity; structural constituent of cytoskeleton; DNA binding; cytoskeletal protein binding; transcription factor activity | nucleobase, nucleoside, nucleotide and nucleic acid metabolic process; protein metabolic process |
| YPEL5 | Protein yippee-like 5 | oxidoreductase activity | respiratory electron transport chain; cellular amino acid and derivative metabolic process; lipid metabolic process; protein metabolic process |
| RIBC1 | RIB43A-like with coiled-coils protein 1 | structural constituent of cytoskeleton | unclear biological function |
| HSPB2 | Heat shock protein beta-2 | structural molecule activity | immune system process; muscle contraction; visual perception; sensory perception; protein metabolic process; response to stress |
| PRUNE | Protein prune homolog | unclear molecular function | unclear biological function |
| PJA2 | E3 ubiquitin-protein ligase Praja2 | DNA binding;  transcription factor activity | unclear biological function |
| KSR2 | Kinase suppressor of Ras 2 | kinase activity;  transmembrane receptor protein serine/threonine kinase activity;  transmembrane receptor protein tyrosine kinase activity;  transmembrane receptor protein kinase activity | female gamete generation; immune system process;  induction of apoptosis; negative regulation of apoptosis;  cell surface receptor linked signal transduction; intracellular signaling cascade; protein metabolic process; cell cycle; signal transduction; cellular component morphogenesis;  response to stress |
| UNK | RING finger protein unkempt homolog | uncelar molecular function | unclear biological function |
| APIP | APAF1-interacting protein | racemase and epimerase activity | carbohydrate metabolic process |
| HK2 | Hexokinase-2 | kinase activity | carbohydrate metabolic process |
| BLM | Bloom syndrome protein | DNA helicase activity; hydrolase activity, acting on ester bonds; nucleic acid binding | mitosis; nucleobase, nucleoside, nucleotide and nucleic acid metabolic process; mitosis; chromosome segregation |
| PSMC3 | 26S protease regulatory subunit 6A | hydrolase activity | protein metabolic process |
| NCOA5 | Nuclear receptor coactivator 5 | receptor activity | nucleobase, nucleoside, nucleotide and nucleic acid metabolic process |
| PRPS2 | Ribose-phosphate pyrophosphokinase 2 | kinase activity; ligase activity | nucleobase, nucleoside, nucleotide and nucleic acid metabolic process |
| APEH | Acylamino-acid-releasing enzyme | peptidase activity | protein metabolic process |
| EPHA4 | Ephrin type-A receptor 4 | kinase activity; transmembrane receptor protein tyrosine kinase activity; transmembrane receptor protein kinase activity; receptor binding | female gamete generation; immune system process; negative regulation of apoptosis; cell cycle; cell surface receptor linked signal transduction; intracellular signaling cascade; cell-cell signaling; cell-cell adhesion; protein metabolic process; cell motion; cell cycle; signal transduction; cell-cell signaling; cell-cell adhesion; ectoderm development; mesoderm development; embryonic development;  nervous system development |
| DNAJB2 | DnaJ homolog subfamily B member 2 | unclear molecular function | immune system process; protein metabolic process; response to stress |
| CSDE1 | Cold shock domain-containing protein E1 | uncelar molecular function | unclear biological function |
| PTER | Phosphotriesterase-related protein | hydrolase activity | unclear biological function |
| RFT1 | Protein RFT1 homolog | Molecular function unclissified | unclear biological function |
| HSDL2 | Hydroxysteroid dehydrogenase-like protein 2 | oxidoreductase activity | visual perception; sensory perception; lipid metabolic process |
| ACTR1A | Alpha-centractin | structural constituent of cytoskeleton | intracellular protein transport; exocytosis; endocytosis; mitosis; cytokinesis; mitosis; cellular component morphogenesis |
| DLD | Dihydrolipoyl dehydrogenase, mitochondrial | oxidoreductase activity | immune system process; respiratory electron transport chain; apoptosis; ferredoxin metabolic process; oxygen and reactive oxygen species metabolic process |
| OMD | Osteomodulin | receptor activity | immune system process; visual perception;sensory perception; cell surface receptor linked signal transduction; cell-cell adhesion; signal transduction; cell-cell adhesion; mesoderm development |
| ASPN | Asporin | receptor activity | immune system process; cell surface receptor linked signal transduction; cell-cell adhesion; signal transduction; mesoderm development; skeletal system development |
| OGN | Mimecan | receptor activity | immune system process; visual perception; sensory perception; cell surface receptor linked signal transduction; cell-cell adhesion; signal transduction; cell-cell adhesion; mesoderm development |
| MYCN | N-myc proto-oncogene protein | DNA binding; transcription factor activity | induction of apoptosis; cell cycle; intracellular signaling cascade; nucleobase, nucleoside, nucleotide and nucleic acid metabolic process; cell cycle; signal transduction |
| PRKCA | Protein kinase C alpha type | kinase activity; calcium ion binding; calmodulin binding; calcium-dependent phospholipid binding | intracellular signaling cascade; protein metabolic process; signal transduction |
| ZNF536 | Zinc finger protein 536 | DNA binding; transcription factor activity | nucleobase, nucleoside, nucleotide and nucleic acid metabolic process |
| LRRC8A | Leucine-rich repeat-containing protein 8A | adenylate cyclase activity; receptor binding; kinase regulator activity | immune system process; cell surface receptor linked signal transduction; intracellular signaling cascade; nucleobase, nucleoside, nucleotide and nucleic acid metabolic process; signal transduction |
| LYRM1 | LYR motif-containing protein 1 | Unclear molecular function | unclear biological function |
| MTSS1 | Metastasis suppressor protein 1 | structural constituent of cytoskeleton | cell cycle; cell adhesion; cell motion; cell cycle; cell adhesion; ectoderm development; nervous system development |
| PHF23 | PHD finger protein 23 | unclear molecular function | unclear biological function |
| TCP11 | T-complex protein 11 homolog | receptor activity | spermatogenesis |
| ABCG8 | ATP-binding cassette sub-family G member 8 | ATPase activity, coupled to transmembrane movement of substances; transmembrane transporter activity; anion channel activity | immune system process; anion transport; lipid transport; oxygen and reactive oxygen species metabolic process; lipid metabolic process; response to stress |
| ARL4C | ADP-ribosylation factor-like protein 4C | GTPase activity; protein binding | intracellular protein transport; vesicle-mediated transport; signal transduction |
| STXBP1 | Syntaxin-binding protein 1 | unclear molecular function | neurotransmitter secretion;  intracellular protein transport; exocytosis; synaptic transmission; cell-cell signaling |
| SPTB | Spectrin beta chain, erythrocyte | unclear molecular function | unclear biological function. |
| ME3 | NADP-dependent malic enzyme, mitochondrial | oxidoreductase activity; acyltransferase activity; carboxy-lyase activity | tricarboxylic acid cycle; carbohydrate metabolic process; cellular amino acid and derivative metabolic process |
| FRMPD4 | FERM and PDZ domain-containing protein 4 | unclear molecular function | unclear biological function |
| FSTL4 | Follistatin-related protein 4 | protein binding | unclear biological function |
| SAP30L | Histone deacetylase complex subunit SAP30L | nucleic acid binding; chromatin binding | nucleobase, nucleoside, nucleotide and nucleic acid metabolic process |
| RSPH1 | Radial spoke head 1 homolog | kinase activity | cell surface receptor linked signal transduction; signal transduction |
| S100A2 | Protein S100-A2 | calcium ion binding; receptor binding; calmodulin binding | immune response; macrophage activation; cell cycle; intracellular signaling cascade; cell motion; cell cycle; signal transduction; response to stimulus |
| INTS3 | Integrator complex subunit 3 | Molecular function unclissified | Unclear biological function |
| DNAH3 | Dynein heavy chain 3, axonemal | microtubule motor activity; hydrolase activity; structural constituent of cytoskeleton | spermatogenesis; fertilization; intracellular protein transport; vesicle-mediated transport; mitosis; cell motion; mitosis; chromosome segregation; cellular component morphogenesis |
| CDH3 | Cadherin-3 | G-protein coupled receptor activity; calcium ion binding | visual perception; sensory perception of sound; sensory perception; cell surface receptor linked signal transduction; cell-cell adhesion; cell motion; signal transduction; cellular component morphogenesis; ectoderm development; mesoderm development; embryonic development; nervous system development; heart development; muscle organ development |
| TSPAN1 | Tetraspanin-1 | receptor activity; receptor binding | gamete generation; immune system process; neurological system process; signal transduction; cell-cell adhesion; cell motion; signal transduction; blood coagulation |
| PRDX1 | Peroxiredoxin-1 | oxidoreductase activity; peroxidase activity | immune system process; oxygen and reactive oxygen species metabolic process |
| ITIH3 | Inter-alpha-trypsin inhibitor heavy chain H3 | protein binding; peptidase inhibitor activity | protein metabolic process |
| SYNE2 | Nesprin-2 | unclear molecular function | unclear biological function |
| MYO9A | Myosin-IXa | motor activity; structural constituent of cytoskeleton; protein binding; small GTPase regulator activity | muscle contraction; sensory perception of sound; sensory perception; intracellular protein transport; vesicle-mediated transport; mitosis;  intracellular signaling cascade; cytokinesis; cell motion; mitosis; signal transduction; cellular component morphogenesis; mesoderm development;  muscle organ development |
| ENOX2 | Protein disulfide-thiol oxidoreductase | oxidoreductase activity; hydrolase activity, acting on ester bonds; nucleic acid binding | respiratory electron transport chain; metabolic process |
| RPS19 | 40S ribosomal protein S19 | structural constituent of ribosome; nucleic acid binding | protein metabolic process |
| CAST | Calpastatin | protein binding; peptidase inhibitor activity | protein metabolic process |
| SPP1 | Osteopontin | receptor binding | immune system process; cell adhesion; cell adhesion; cellular component morphogenesis |
| PPIG | Peptidyl-prolyl cis-trans isomerase G | isomerase activity | immune system process; intracellular protein transport; nuclear transport; protein metabolic process |
| ITIH4 | 35 kDa inter-alpha-trypsin inhibitor heavy chain H4 | protein binding; peptidase inhibitor activity | protein metabolic process |
| ACAD9 | Acyl-CoA dehydrogenase family member 9, mitochondrial | oxidoreductase activity; transferase activity | respiratory electron transport chain; acyl-CoA metabolic process; nitrogen compound metabolic process; lipid metabolic process |
| CAMTA1 | Calmodulin-binding transcription activator 1 | DNA binding; transcription factor activity | intracellular signaling cascade; nucleobase, nucleoside, nucleotide and nucleic acid metabolic process; signal transduction |
| RNF220 | RING finger protein 220 | unclear molecular function | unclear biological function |

## Table S3 - The list and characteristics of genes up-regulated in tumours group no. 1 in comparison to tumours group no. 3.

Symbol, name and characteristics of up-regulated genes in tumours group no. 1 in comparison to tumours group no. 3. The biological processes and molecular functions of the genes are based on the PANTHER database (www.pantherdb.org). Only significantly regulated genes were included in this table (p<0.01). The genes significantly up-regulated in comparison to the tumour group no. 2 are bolded.

| **Gene symbol** | **Gene name** | **Molecular function** | **Biological process** |
| --- | --- | --- | --- |
| NELL2 | Protein kinase C-binding protein NELL2 | calcium ion binding; calmodulin binding; calcium-dependent phospholipid binding | signal transduction; cell adhesion; signal transduction; ectoderm development; nervous system development |
| GGNBP2 | Gametogenetin-binding protein 2 | unclear molecular function | unclear biological function. |
| ACSS1 | Acetyl-coenzyme A synthetase 2-like, mitochondrial | ligase activity | immune system process; coenzyme metabolic process; lipid metabolic process |
| UBR1 | E3 ubiquitin-protein ligase UBR1 | ubiquitin-protein ligase activity | protein metabolic process |
| PREX2 | Phosphatidylinositol 3,4,5-trisphosphate-dependent Rac exchanger 2 protein | receptor activity; protein binding; small GTPase regulator activity; guanyl-nucleotide exchange factor activity | unclear biological function |
| SPAG11B | Sperm-associated antigen 11B | unclear molecular function | unclear biological function |
| WBP11 | WW domain-binding protein 11 | unclear molecular function | unclear biological function |
| ZFP37 | Zinc finger protein 37 homolog | DNA binding; transcription factor activity | spermatogenesis; nucleobase, nucleoside, nucleotide and nucleic acid metabolic process |
| **ATP2C1** | Calcium-transporting ATPase type 2C member 1 | hydrolase activity; cation transmembrane transporter activity; ion channel activity | cation transport; lipid transport; lipid metabolic process; cellular calcium ion homeostasis |
| L2HGDH | L-2-hydroxyglutarate dehydrogenase, mitochondrial | oxidoreductase activity | respiratory electron transport chain; cellular amino acid and derivative metabolic process; lipid metabolic process; protein metabolic process |
| **SIGLEC11** | Sialic acid-binding Ig-like lectin 11 | receptor activity; structural constituent of myelin sheath; receptor binding | B cell mediated immunity; cell surface receptor linked signal transduction; cell-cell adhesion; signal transduction; cell-cell adhesion; response to stimulus |
| UTP18 | U3 small nucleolar RNA-associated protein 18 homolog | unclear molecular function | unclear biological function |
| KHDRBS3 | KH domain-containing, RNA-binding, signal transduction-associated protein 3 | RNA splicing factor activity, transesterification mechanism; DNA binding; RNA binding; transcription factor activity; transcription cofactor activity | spermatogenesis; nucleobase, nucleoside, nucleotide and nucleic acid metabolic process |
| SPATS2 | Spermatogenesis-associated serine-rich protein 2 | unclear molecular function | unclear biological function |
| SPEN | Msx2-interacting protein | RNA splicing factor activity, transesterification mechanism; RNA binding | nucleobase, nucleoside, nucleotide and nucleic acid metabolic process |
| NIPSNAP3A | Protein NipSnap homolog 3A | unclear molecular function | intracellular protein transport; vesicle-mediated transport |
| MRPS18C | 28S ribosomal protein S18c, mitochondrial | structural constituent of ribosome; nucleic acid binding | protein metabolic process |
| ELAVL3 | ELAV-like protein 3 | RNA splicing factor activity, transesterification mechanism; RNA binding | signal transduction; nucleobase, nucleoside, nucleotide and nucleic acid metabolic process; protein metabolic process; signal transduction; ectoderm development; nervous system development |
| TRAP1 | Heat shock protein 75 kDa, mitochondrial | unclear molecular function | immune system process; protein metabolic process; response to stress |
| HRSP12 | Ribonuclease UK114 | unclear molecular function | protein metabolic process |
| ARHGEF3 | Rho guanine nucleotide exchange factor 3 | protein binding; small GTPase regulator activity; guanyl-nucleotide exchange factor activity | amino acid transport; intracellular protein transport; endocytosis; apoptosis; cell cycle; signal transduction; cell adhesion; cellular amino acid and derivative metabolic process; cell cycle; signal transduction; cell adhesion |
| C16orf63 | LisH domain-containing protein C16orf63 | unclear molecular function | unclear biological function |
| WDR85 | WD repeat-containing protein 85 | receptor activity | intracellular protein transport; peroxisomal transport; nucleobase, nucleoside, nucleotide and nucleic acid metabolic process; organelle organization; establishment or maintenance of chromatin architecture |
| PIK3CB | Phosphatidylinositol-4,5-bisphosphate 3-kinase catalytic subunit beta isoform | kinase activity | intracellular protein transport; endocytosis; negative regulation of apoptosis; cell surface receptor linked signal transduction; intracellular signaling cascade; phosphate metabolic process; lipid metabolic process; signal transduction |
| **GPR155** | Integral membrane protein GPR155 | receptor activity | unclear biological function |
| **HERC4** | Probable E3 ubiquitin-protein ligase HERC4 | ubiquitin-protein ligase activity | protein metabolic process; ectoderm development; mesoderm development; skeletal system development; nervous system development |
| COBLL1 | Cordon-bleu protein-like 1 | unclear molecular function | ectoderm development; nervous system development |
| FETUB | Fetuin-B | protein binding; peptidase inhibitor activity | immune system process; protein metabolic process; mesoderm development; skeletal system development |
| **RDH5** | 11-cis retinol dehydrogenase | oxidoreductase activity | visual perception; sensory perception; lipid metabolic process |
| **TMEM98** | Transmembrane protein 98 | unclear molecular function | unclear biological function |
| PARP4 | Poly [ADP-ribose] polymerase 4 | protein binding; peptidase inhibitor activity | protein metabolic process |
| IL21R | Interleukin-21 receptor | cytokine receptor activity | natural killer cell activation; cell surface receptor linked signal transduction; intracellular signaling cascade; cell-cell signaling; signal transduction; cell-cell signaling; mesoderm development; hemopoiesis; response to stimulus |
| MEF2B | Myocyte-specific enhancer factor 2B | DNA binding; transcription factor activity | nucleobase, nucleoside, nucleotide and nucleic acid metabolic process; mesoderm development; embryonic development; muscle organ development |
| SARS | Seryl-tRNA synthetase, cytoplasmic | aminoacyl-tRNA ligase activity; RNA binding | protein metabolic process |
| IL5RA | Interleukin-5 receptor subunit alpha | cytokine receptor activity; receptor binding | B cell mediated immunity; cell surface receptor linked signal transduction; intracellular signaling cascade; cell-cell signaling; signal transduction; cell-cell signaling; ectoderm development;  mesoderm development; nervous system development; hemopoiesis; response to stimulus |
| STAU1 | Double-stranded RNA-binding protein Staufen homolog 1 | hydrolase activity; deaminase activity; DNA binding; RNA binding; protein binding; kinase activator activity; kinase regulator activity | spermatogenesis; response to interferon-gamma; neurological system process; apoptosis; cell cycle; nucleobase, nucleoside, nucleotide and nucleic acid metabolic process; protein metabolic process; cell cycle; anterior/posterior axis specification; response to stimulus; RNA localization |
| SMG6 | Telomerase-binding protein EST1A | DNA binding | mitosis; mitosis; chromosome segregation |
| **ATF6** | Processed cyclic AMP-dependent transcription factor ATF-6 alpha | DNA binding; transcription factor activity | immune system process; neurological system process; signal transduction; nucleobase, nucleoside, nucleotide and nucleic acid metabolic process; signal transduction; ectoderm development; nervous system development; response to stress |
| SPAG11A | Sperm-associated antigen 11A | unclear molecular function | unclear biological function |
| **SIGLEC10** | Sialic acid-binding Ig-like lectin 10 | receptor activity; structural constituent of myelin sheath; receptor binding | B cell mediated immunity; cell surface receptor linked signal transduction; cell-cell adhesion; signal transduction;  cell-cell adhesion; response to stimulus |
| CRYZL1 | Quinone oxidoreductase-like protein 1 | oxidoreductase activity | apoptosis;carbohydrate metabolic process |
| NUP98 | Nuclear pore complex protein Nup96 | transmembrane transporter activity | intracellular protein transport; nuclear transport; nucleobase, nucleoside, nucleotide and nucleic acid metabolic process; RNA localization |
| SLMAP | Sarcolemmal membrane-associated protein | unclear molecular function | unclear biological function |
| AMBRA1 | Activating molecule in BECN1-regulated autophagy protein 1 | unclear molecular function | unclear biological function |
| MLL2 | Histone-lysine N-methyltransferase MLL2 | methyltransferase activity; DNA binding | nucleobase, nucleoside, nucleotide and nucleic acid metabolic process; organelle organization; establishment or maintenance of chromatin architecture |
| ABCA1 | ATP-binding cassette sub-family A member 1 | ATPase activity, coupled to transmembrane movement of substances; transmembrane transporter activity | lipid transport; lipid metabolic process |
| WDR55 | WD repeat-containing protein 55 | unclear molecular function | unclear biological function |
| NIPSNAP3B | Protein NipSnap homolog 3B | unclear molecular function | intracellular protein transport; vesicle-mediated transport |
| AQP9 | Aquaporin-9 | transmembrane transporter activity | transport; lipid metabolic process |
| COG5 | Conserved oligomeric Golgi complex subunit 5 | unclear molecular function | intracellular protein transport |
| SPIB | Transcription factor Spi-B | DNA binding; receptor binding; transcription factor activity | B cell mediated immunity; macrophage activation; cell cycle; cell surface receptor linked signal transduction; intracellular signaling cascade; nucleobase, nucleoside, nucleotide and nucleic acid metabolic process; cell cycle; signal transduction; endoderm development; mesoderm development; hemopoiesis; cellular defense response |
| IL13RA2 | Interleukin-13 receptor alpha-2 chain | cytokine receptor activity; receptor binding | B cell mediated immunity; cell surface receptor linked signal transduction; intracellular signaling cascade; cell-cell signaling; signal transduction; cell-cell signaling; ectoderm development; mesoderm development; nervous system development; hemopoiesis; response to stimulus |
| TOB1 | Protein Tob1 | unclear molecular function | cell cycle; cell surface receptor linked signal transduction; intracellular signaling cascade; cell cycle; signal transduction |
| ACIN1 | Apoptotic chromatin condensation inducer in the nucleus | nucleic acid binding; chromatin binding | apoptosis |
| NASP | Nuclear autoantigenic sperm protein | unclear molecular function | intracellular protein transport; nuclear transport; nucleobase, nucleoside, nucleotide and nucleic acid metabolic process; organelle organization; establishment or maintenance of chromatin architecture |
| ACSM3 | Acyl-coenzyme A synthetase ACSM3, mitochondrial | ligase activity | immune system process; coenzyme metabolic process; lipid metabolic process |
| CES2 | Carboxylesterase 2 | hydrolase activity, acting on ester bonds; receptor binding | immune system process; neurological system process; meiosis; signal transduction; synaptic transmission; cell adhesion; vitamin metabolic process; lipid metabolic process; meiosis; signal transduction; cell-cell signaling; cell adhesion; ectoderm development; nervous system development; response to toxin |
| GABRG2 | Gamma-aminobutyric acid receptor subunit gamma-2 | acetylcholine receptor activity; GABA receptor activity; ligand-gated ion channel activity | muscle contraction; neurological system process; cation transport; anion transport; cell surface receptor linked signal transduction; synaptic transmission; signal transduction; cell-cell signaling |
| CLIP1 | CAP-Gly domain-containing linker protein 1 | structural constituent of cytoskeleton; cytoskeletal protein binding | intracellular protein transport; vesicle-mediated transport; mitosis; protein metabolic process; mitosis; cellular component morphogenesis |
|  | Androgen-induced protein 1;AIG1 | unclear molecular function | unclear biological function |
| RPS14 | 40S ribosomal protein S14 | structural constituent of ribosome; nucleic acid binding | protein metabolic process |
| USP20 | Ubiquitin carboxyl-terminal hydrolase 20 | peptidase activity; ubiquitin-protein ligase activity; RNA splicing factor activity, transesterification mechanism; RNA binding | gamete generation; nucleobase, nucleoside, nucleotide and nucleic acid metabolic process; protein metabolic process |
| RNF115 | RING finger protein 115 | unclear molecular function | unclear biological function |
| GAPDH | Glyceraldehyde-3-phosphate dehydrogenase | oxidoreductase activity | carbohydrate metabolic process |
| TGM7 | Protein-glutamine gamma-glutamyltransferase Z | acyltransferase activity | protein metabolic process |
| POLDIP3 | Polymerase delta-interacting protein 3 | RNA binding | nucleobase, nucleoside, nucleotide and nucleic acid transport; nucleobase, nucleoside, nucleotide and nucleic acid metabolic process |
| ARMC6 | Armadillo repeat-containing protein 6 | unclear molecular function | unclear biological function |
| DDB2 | DNA damage-binding protein 2 | DNA binding | nucleobase, nucleoside, nucleotide and nucleic acid metabolic process |
| SSR3 | Translocon-associated protein subunit gamma | unclear molecular function | intracellular protein transport |
| SLC22A11 | Solute carrier family 22 member 11 | ATPase activity, coupled to transmembrane movement of substances; ligase activity; carbohydrate transmembrane transporter activity; cation transmembrane transporter activity | cation transport; anion transport; extracellular transport; carbohydrate transport; carbohydrate metabolic process |
| SCLY | Selenocysteine lyase | lyase activity | sulfur metabolic process; cellular amino acid and derivative metabolic process |
| **SPLUNC3** | Short palate, lung and nasal epithelium carcinoma-associated protein 3 | carbohydrate transmembrane transporter activity; transmembrane transporter activity | macrophage activation; lipid transport; lipid metabolic process; response to stress; defense response to bacterium |
| SOS2 | Son of sevenless homolog 2 | protein binding; small GTPase regulator activity; guanyl-nucleotide exchange factor activity | mitosis; cell surface receptor linked signal transduction; intracellular signaling cascade; mitosis; signal transduction |
| DONSON | Protein downstream neighbor of Son | unclear molecular function | unclear biological function |
| PIGU | Phosphatidylinositol glycan anchor biosynthesis class U protein | unclear molecular function | intracellular protein transport |
| HERC3 | Probable E3 ubiquitin-protein ligase HERC3 | ubiquitin-protein ligase activity | protein metabolic process; ectoderm development; mesoderm development; skeletal system development; nervous system development |
| ZNF567 | Zinc finger protein 567 | DNA binding; transcription factor activity | nucleobase, nucleoside, nucleotide and nucleic acid metabolic process |
| SPOCK3 | Testican-3 | calcium ion binding;protein binding; peptidase inhibitor activity | unclear biological function |
| IK | Protein Red | receptor binding | immune response; signal transduction; cell-cell signaling; signal transduction; cell-cell signaling; response to stimulus |
| **ABHD7** | Abhydrolase domain-containing protein 7 | peptidase activity | immune system process; protein metabolic process; response to toxin |
| C8orf47 | Uncharacterized protein C8orf47 | unclear molecular function | unclear biological function |
| PRKAR2B | cAMP-dependent protein kinase type II-beta regulatory subunit | protein binding; kinase regulator activity | signal transduction; protein metabolic process; signal transduction |
| APP | C31 | receptor binding | intracellular protein transport; signal transduction; signal transduction |
| ACSM1 | Acyl-coenzyme A synthetase ACSM1, mitochondrial | ligase activity | immune system process; coenzyme metabolic process; lipid metabolic process |
| HECTD1 | E3 ubiquitin-protein ligase HECTD1 | ubiquitin-protein ligase activity | protein metabolic process; ectoderm development; mesoderm development; skeletal system development; nervous system development |
| CDKN3 | Cyclin-dependent kinase inhibitor 3 | hydrolase activity, acting on ester bonds;phosphatase activity | cell cycle; protein metabolic process; cell cycle |
| C5orf44 | UPF0533 protein C5orf44 | unclear molecular function | unclear biological function |
| SLC36A1 | Proton-coupled amino acid transporter 1 | unclear molecular function | unclear biological function |
| ZNF786 | Zinc finger protein 786 | DNA binding; transcription factor activity | nucleobase, nucleoside, nucleotide and nucleic acid metabolic process |
| FSHR | Follicle-stimulating hormone receptor; | receptor activity | cell surface receptor linked signal transduction; signal transduction |
| KIF16B | Kinesin-like protein KIF16B | microtubule motor activity; structural constituent of cytoskeleton | intracellular protein transport; vesicle-mediated transport; mitosis; meiosis; cytokinesis; mitosis; meiosis; chromosome segregation; cellular component morphogenesis; cellular component morphogenesis; cellular component morphogenesis |
| PQLC1 | PQ-loop repeat-containing protein 1 | unclear molecular function | unclear biological function |
| CACNA2D3 | Voltage-dependent calcium channel subunit delta-3 | cation transmembrane transporter activity; voltage-gated calcium channel activity; cation channel activity | muscle contraction; neurological system process; cation transport; intracellular protein transport; intracellular signaling cascade; signal transduction |
| OAS1 | 2'-5'-oligoadenylate synthetase 1 | nucleotidyltransferase activity; nucleic acid binding | response to interferon-gamma; nucleobase, nucleoside, nucleotide and nucleic acid metabolic process;response to stimulus |
| OAS3 | 2'-5'-oligoadenylate synthetase 3 | nucleotidyltransferase activity; nucleic acid binding | response to interferon-gamma; nucleobase, nucleoside, nucleotide and nucleic acid metabolic process; response to stimulus |
| TGM5 | Protein-glutamine gamma-glutamyltransferase 5 | acyltransferase activity | protein metabolic process |
| SPC24 | Kinetochore protein Spc24 | unclear molecular function | unclear biological function |
| CYP4A22 | Cytochrome P450 4A22 | oxidoreductase activity | respiratory electron transport chain; lipid metabolic process |
| IDH3A | Isocitrate dehydrogenase [NAD] subunit alpha, mitochondrial | oxidoreductase activity | tricarboxylic acid cycle; carbohydrate metabolic process; cellular amino acid and derivative metabolic process |
| NP60 | Nuclear protein NP60 | oxidoreductase activity | cellular amino acid and derivative metabolic process |
| ODZ1 | Teneurin-1 | hydrolase activity, hydrolyzing O-glycosyl compounds; receptor activity; receptor binding | intracellular protein transport; intracellular signaling cascade; cell-cell adhesion; protein metabolic process;  signal transduction; anterior/posterior axis specification; ectoderm development; mesoderm development; nervous system development |
| PPP1R12B | Protein phosphatase 1 regulatory subunit 12B | protein binding; phosphatase regulator activity | protein metabolic process |
| FECH | Ferrochelatase, mitochondrial | lyase activity | porphyrin metabolic process |
| REV3L | DNA polymerase zeta catalytic subunit | DNA-directed DNA polymerase activity; hydrolase activity, acting on ester bonds; nucleic acid binding | cell cycle; nucleobase, nucleoside, nucleotide and nucleic acid metabolic process; cell cycle |
| IPO8 | Importin-8 | GTPase activity; transmembrane transporter activity; protein binding | intracellular protein transport; nuclear transport |
| CNOT7 | CCR4-NOT transcription complex subunit 7 | DNA binding; transcription factor activity | nucleobase, nucleoside, nucleotide and nucleic acid metabolic process |
| KCNC2 | Potassium voltage-gated channel subfamily C member 2 | cation transmembrane transporter activity; voltage-gated potassium channel activity; cation channel activity | muscle contraction; blood circulation; neuronal action potential propagation; cation transport; signal transduction; synaptic transmission; signal transduction; cell-cell signaling |
| **HYOU1** | Hypoxia up-regulated protein 1 | unclear molecular function | immune system process; protein metabolic process; response to stress |
| ART4 | Ecto-ADP-ribosyltransferase 4 | transferase activity, transferring glycosyl groups | protein metabolic process |
| IGFBP7 | Insulin-like growth factor-binding protein 7 | unclear molecular function | unclear biological function |
| ZNF382 | Zinc finger protein 382 | DNA binding; transcription factor activity | nucleobase, nucleoside, nucleotide and nucleic acid metabolic process |
| KANK2 | KN motif and ankyrin repeat domain-containing protein 2 | unclear molecular function | unclear biological function |
| C3orf59 | Uncharacterized protein C3orf59 | unclear molecular function | unclear biological function |
| IRF3 | Interferon regulatory factor 3 | DNA binding; transcription factor activity | response to interferon-gamma; nucleobase, nucleoside, nucleotide and nucleic acid metabolic process; response to stimulus |
| ACSM5 | Acyl-coenzyme A synthetase ACSM5, mitochondrial | ligase activity | immune system process; coenzyme metabolic process; lipid metabolic process |
| ADH4 | Alcohol dehydrogenase 4 | oxidoreductase activity | apoptosis; carbohydrate metabolic process |
| CYP4B1 | Cytochrome P450 4B1 | oxidoreductase activity | respiratory electron transport chain; lipid metabolic process |
| FAM151B | Protein FAM151B | unclear molecular function | unclear biological function |
| SACS | Sacsin | unclear molecular function | unclear biological function |
| RBCK1 | RanBP-type and C3HC4-type zinc finger-containing protein 1 | ubiquitin-protein ligase activity | immune system process; protein metabolic process |
| AKAP2 | A-kinase anchor protein 2 | unclear molecular function | signal transduction; signal transduction |
| **ARHGAP15** | Rho GTPase-activating protein 15 | protein binding; small GTPase regulator activity | intracellular signaling cascade; signal transduction |
| PIGG | GPI ethanolamine phosphate transferase 2 | unclear molecular function | protein metabolic process |
| CALB1 | Calbindin | calcium ion binding | intracellular signaling cascade; signal transduction; cellular calcium ion homeostasis |
| MTMR4 | Myotubularin-related protein 4 | unclear molecular function | spermatogenesis; intracellular protein transport; vesicle-mediated transport; lipid metabolic process |
| AUTS2 | Autism susceptibility gene 2 protein | unclear molecular function | unclear biological function |
| **NR6A1** | Nuclear receptor subfamily 6 group A member 1 | ligand-dependent nuclear receptor activity; DNA binding; transcription factor activity | spermatogenesis; intracellular signaling cascade; nucleobase, nucleoside, nucleotide and nucleic acid metabolic process; signal transduction |
| ARF6 | ADP-ribosylation factor 6 | GTPase activity; protein binding | intracellular protein transport; vesicle-mediated transport; signal transduction; signal transduction |
| THUMPD3 | THUMP domain-containing protein 3 | unclear molecular function | unclear biological function |
| MRPL37 | 39S ribosomal protein L37, mitochondrial | structural constituent of ribosome; nucleic acid binding | protein metabolic process |
| RNFT2 | Ring finger and transmembrane domain-containing protein 2 | unclear molecular function | unclear biological function |
| ZNF239 | Zinc finger protein 239 | DNA binding; transcription factor activity | nucleobase, nucleoside, nucleotide and nucleic acid metabolic process |
| CUTC | Copper homeostasis protein cutC homolog | cation transmembrane transporter activity | cation transport |
| DTNB | Dystrobrevin beta | unclear molecular function | unclear biological function |
| CPNE8 | Copine-8 | unclear molecular function | intracellular protein transport |
| HRK | Activator of apoptosis harakiri | unclear molecular function | unclear biological function |
| NSD1 | Histone-lysine N-methyltransferase, H3 lysine-36 and H4 lysine-20 specific | methyltransferase activity; DNA binding | nucleobase, nucleoside, nucleotide and nucleic acid metabolic process; organelle organization; establishment or maintenance of chromatin architecture |
| FAIM | Fas apoptotic inhibitory molecule 1 | unclear molecular function | unclear biological function |
| COL1A2 | Collagen alpha-2(I) chain | receptor activity; extracellular matrix structural constituent; receptor binding | complement activation; macrophage activation; blood circulation; carbohydrate transport; signal transduction; cell-cell signaling; cell-cell adhesion; carbohydrate metabolic process; lipid metabolic process; signal transduction; cell-cell signaling;  cellular component morphogenesis; ectoderm development; mesoderm development; skeletal system development; angiogenesis; regulation of liquid surface tension; response to stimulus |
| **GCSH** | Glycine cleavage system H protein, mitochondrial | hydrolase activity | cellular amino acid and derivative metabolic process |
| BTBD10 | BTB/POZ domain-containing protein 10 | unclear molecular function | protein metabolic process |
| OAS2 | 2'-5'-oligoadenylate synthetase 2 | nucleotidyltransferase activity; nucleic acid binding | response to interferon-gamma; nucleobase, nucleoside, nucleotide and nucleic acid metabolic process; response to stimulus |
| DLD | Dihydrolipoyl dehydrogenase, mitochondrial | oxidoreductase activity | immune system process; respiratory electron transport chain; apoptosis; ferredoxin metabolic process; oxygen and reactive oxygen species metabolic process |
| TCTA | T-cell leukemia translocation-altered gene protein | unclear molecular function | unclear biological function |
| MOBKL3 | Mps one binder kinase activator-like 3 | protein binding; kinase activator activity; kinase regulator activity | mitosis; cytokinesis; mitosis |
| GCH1 | GTP cyclohydrolase 1 | hydrolase activity | coenzyme metabolic process; nucleobase, nucleoside, nucleotide and nucleic acid metabolic process |
| ARID3B | AT-rich interactive domain-containing protein 3B | DNA binding; transcription factor activity | nucleobase, nucleoside, nucleotide and nucleic acid metabolic process |
| EDC3 | Enhancer of mRNA-decapping protein 3 | unclear molecular function | unclear biological function |
| **ZNF331** | Zinc finger protein 331 | DNA binding; transcription factor activity | nucleobase, nucleoside, nucleotide and nucleic acid metabolic process |
| MAPK6 | Mitogen-activated protein kinase 6 | kinase activity | immune system process; mitosis; cell surface receptor linked signal transduction; intracellular signaling cascade; carbohydrate metabolic process; protein metabolic process; cell motion; mitosis; signal transduction; segmentspecification; ectoderm development; mesoderm development; embryonic development; nervous system development; response to stress |
| LSR | Lipolysis-stimulated lipoprotein receptor | unclear molecular function | unclear biological function |
| ANKS1A | Ankyrin repeat and SAM domain-containing protein 1A | unclear molecular function | cell cycle; cell surface receptor linked signal transduction; cell cycle; signal transduction |
| **NR5A1** | Steroidogenic factor 1 | ligand-dependent nuclear receptor activity; DNA binding; transcription factor activity | signal transduction; nucleobase, nucleoside, nucleotide and nucleic acid metabolic process; signal transduction |
| ATP6V0D1 | V-type proton ATPase subunit d 1 | hydrolase activity; cation transmembrane transporter activity; hydrogen ion transmembrane transporter activity | cation transport |
| SYCP3 | Synaptonemal complex protein 3 | unclear molecular function | meiosis |
| TMCC1 | Transmembrane and coiled-coil domains protein 1 | unclear molecular function | unclear biological function |
| CCDC93 | Coiled-coil domain-containing protein 93 | unclear molecular function | unclear biological function |
| SIPA1L2 | Signal-induced proliferation-associated 1-like protein 2 | protein binding; small GTPase regulator activity | cell adhesion |
| FAM40A | Protein FAM40A | unclear molecular function | unclear biological function |
| SP110 | Sp110 nuclear body protein | DNA binding; chromatin binding; receptor binding; transcription factor activity | intracellular signaling cascade; nucleobase, nucleoside, nucleotide and nucleic acid metabolic process; signal transduction; organelle organization; establishment or maintenance of chromatin architecture |
| **PLUNC** | Protein Plunc | carbohydrate transmembrane transporter activity; transmembrane transporter activity | macrophage activation; lipid transport; lipid metabolic process; response to stress; defense response to bacterium |
| DNAJC1 | DnaJ homolog subfamily C member 1 | unclear molecular function | immune system process; cell cycle; protein metabolic process; cell cycle; response to stress |
| WBP4 | WW domain-binding protein 4 | unclear molecular function | unclear biological function |
| CREBBP | CREB-binding protein | acyltransferase activity; DNA binding; chromatin binding; transcription factor activity; transcription cofactor activity | nucleobase, nucleoside, nucleotide and nucleic acid metabolic process |
| CENPC1 | Centromere protein C 1 | nucleic acid binding | mitosis; mitosis; chromosome segregation |
| ATP2A2 | Sarcoplasmic/endoplasmic reticulum calcium ATPase 2 | hydrolase activity; cation transmembrane transporter activity; ion channel activity | cation transport; lipid transport; lipid metabolic process; cellular calcium ion homeostasis |
| WDR34 | WD repeat-containing protein 34 | structural constituent of cytoskeleton | intracellular protein transport; vesicle-mediated transport; cell cycle; nucleobase, nucleoside, nucleotide and nucleic acid metabolic process; cell cycle; cellular component morphogenesis; cellular component morphogenesis; cellular component morphogenesis; RNA localization |
| PPM1L | Protein phosphatase 1L | hydrolase activity, acting on ester bonds; phosphatase activity; protein binding; kinase inhibitor activity; kinase activator activity; kinase regulator activity | cell surface receptor linked signal transduction; intracellular signaling cascade; protein metabolic process; signal transduction |
| USP3 | Ubiquitin carboxyl-terminal hydrolase 3 | peptidase ctivity; ubiquitin-protein ligase activity; RNA splicing factor activity, transesterification mechanism; RNA binding | gamete generation; nucleobase, nucleoside, nucleotide and nucleic acid metabolic process; protein metabolic process |
| REN | Renin | peptidase activity | antigen processing and presentation of peptide or polysaccharide antigen via MHC class II; protein metabolic process; cellular defense response |
| CYP4Z1 | Cytochrome P450 4Z1 | oxidoreductase activity | respiratory electron transport chain; lipid metabolic process |
| **BNIPL** | Bcl-2/adenovirus E1B 19 kDa-interacting protein 2-like protein | unclear molecular function | apoptosis |
| IQGAP2 | Ras GTPase-activating-like protein IQGAP2 | protein binding; small GTPase regulator activity | mitosis; intracellular signaling cascade; cytokinesis; mitosis; signal transduction |
| RPL32 | 60S ribosomal protein L32 | structural constituent of ribosome; nucleic acid binding | protein metabolic process |
| AGXT2L2 | Alanine--glyoxylate aminotransferase 2-like 2 | transaminase activity | visual perception; sensory perception; vitamin biosynthetic process; cellular amino acid and derivative metabolic process |
| SNCA | Alpha-synuclein | structural constituent of cytoskeleton; receptor binding | neurotransmitter secretion; intracellular protein transport; exocytosis;  apoptosis; intracellular signaling cascade; synaptic transmission; signal transduction; cell-cell signaling |
| KCNB1 | Potassium voltage-gated channel subfamily B member 1 | cation transmembrane transporter activity; voltage-gated potassium channel activity; cation channel activity | muscle contraction; blood circulation; neuronal action potential propagation; cation transport; signal transduction; synaptic transmission; signal transduction; cell-cell signaling |
| TBC1D1 | TBC1 domain family member 1 | hydrolase activity; protein binding; small GTPase regulator activity | intracellular protein transport; exocytosis; cellular component morphogenesis; cellular component morphogenesis; cellular component morphogenesis |
| ADAM22 | ADAM 22 | peptidase activity | fertilization; neurological system process; apoptosis; signal transduction; cell-cell adhesion; protein metabolic process; signal transduction; mesoderm development; heart development |
| METTL8 | Methyltransferase-like protein 8 | methyltransferase activity | unclear biological function |
| GTPBP8 | GTP-binding protein 8 | GTPase activity; protein binding | carbohydrate metabolic process; nucleobase, nucleoside, nucleotide and nucleic acid metabolic process; protein metabolic process |
| MYBPC1 | Myosin-binding protein C, slow-type | kinase activity; structural constituent of cytoskeleton; protein binding; small GTPase regulator activity;  guanyl-nucleotide exchange factor activity | muscle contraction; intracellular protein transport; endocytosis; signal transduction; cell adhesion; protein metabolic process; cell motion; signal transduction; cell adhesion; cellular component morphogenesis; cellular component morphogenesis; mesoderm development; cellular component morphogenesis; muscle organ development |
| HSF1 | Heat shock factor protein 1 | DNA binding; transcription factor activity | immune system process; nucleobase, nucleoside, nucleotide and nucleic acid metabolic process; response to stress |
| CHPT1 | Cholinephosphotransferase 1 | transferase activity | lipid metabolic process |
| ZDHHC13 | Probable palmitoyltransferase ZDHHC13 | unclear molecular function | unclear biological function |
| **RFWD2** | E3 ubiquitin-protein ligase RFWD2 | hydrolase activity, acting on ester bonds; ubiquitin-protein ligase activity; RNA splicing factor activity, transesterification mechanism; RNA binding; protein binding; kinase inhibitor activity; kinase regulator activity | nucleobase, nucleoside, nucleotide and nucleic acid metabolic process; protein metabolic process; ectoderm development; nervous system development; RNA localization |
| SLC4A2 | Anion exchange protein 2 | transmembrane transporter activity | ion transport; cellular component morphogenesis |
| EXOC4 | Exocyst complex component 4 | unclear molecular function | intracellular protein transport; exocytosis |
| POLD1 | DNA polymerase delta catalytic subunit | DNA-directed DNA polymerase activity; hydrolase activity, acting on ester bonds; nucleic acid binding | cell cycle; nucleobase, nucleoside, nucleotide and nucleic acid metabolic process; cell cycle |
| WHSC1L1 | Histone-lysine N-methyltransferase NSD3 | methyltransferase activity; DNA binding | nucleobase, nucleoside, nucleotide and nucleic acid metabolic process; organelle organization; establishment or maintenance of chromatin architecture |
| TPM3 | Tropomyosin alpha-3 chain | motor activity; structural constituent of cytoskeleton | muscle contraction; cell motion; cellular component morphogenesis; mesoderm development; muscle organ development |
| KIAA1012 | Protein TRS85 homolog | unclear molecular function | transport |
| CHST11 | Carbohydrate sulfotransferase 11 | transferase activity | sulfur metabolic process |
| ZNF485 | Zinc finger protein 485 | DNA binding; transcription factor activity | nucleobase, nucleoside, nucleotide and nucleic acid metabolic process |
| ATF2 | Cyclic AMP-dependent transcription factor ATF-2 | DNA binding; transcription factor activity | intracellular signaling cascade; nucleobase, nucleoside, nucleotide and nucleic acid metabolic process; signal transduction |
| MAP2K5 | Dual specificity mitogen-activated protein kinase kinase 5 | kinase activity | phosphate metabolic process; protein metabolic process |
| ADH5 | Alcohol dehydrogenase class-3 | oxidoreductase activity | apoptosis; carbohydrate metabolic process |
| SLC36A3 | Proton-coupled amino acid transporter 3 | unclear molecular function | unclear biological function |
| GPR143 | G-protein coupled receptor 143 | unclear molecular function | unclear biological function |
| CYP4X1 | Cytochrome P450 4X1 | oxidoreductase activity | respiratory electron transport chain; lipid metabolic process |
| PLEKHM3 | Pleckstrin homology domain-containing family M member 3 | unclear molecular function | unclear biological function |
| MYLC2B | Myosin regulatory light chain MRLC2 | structural constituent of cytoskeleton; calcium ion binding; calmodulin binding | muscle contraction; mesoderm development; muscle organ development |
| WWOX | WW domain-containing oxidoreductase | oxidoreductase activity | visual perception; sensory perception; lipid metabolic process |
| ACSM2B | Acyl-coenzyme A synthetase ACSM2B, mitochondrial | ligase activity | immune system process; coenzyme metabolic process; lipid metabolic process |
| GRB14 | Growth factor receptor-bound protein 14 | unclear molecular function | cell surface receptor linked signal transduction; signal transduction |
| **MYL6B** | Myosin light chain 6B | structural constituent of cytoskeleton; calcium ion binding; calmodulin binding | muscle contraction |
| **MYL6** | Myosin light polypeptide 6 | structural constituent of cytoskeleton; calcium ion binding; calmodulin binding | muscle contraction |
| FRY | Protein furry homolog | unclear molecular function | unclear biological function |
| RUFY2 | RUN and FYVE domain-containing protein 2 | DNA binding;protein binding; small GTPase regulator activity; transcription factor activity | intracellular protein transport; nucleobase, nucleoside, nucleotide and nucleic acid metabolic process |
| SLC22A12 | Solute carrier family 22 member 12 | ATPase activity, coupled to transmembrane movement of substances; ligase activity; carbohydrate transmembrane transporter activity; cation transmembrane transporter activity | cation transport; anion transport; extracellular transport; carbohydrate transport; carbohydrate metabolic process |
| PCDH1 | Protocadherin-1 | G-protein coupled receptor activity; calcium ion binding | visual perception; sensory perception of sound; sensory perception; cell surface receptor linked signal transduction;  cell-cell adhesion; cell motion; signal transduction;  cellular component morphogenesis; ectoderm development; mesoderm development; embryonic development; nervous system development; heart development; muscle organ development |
| RUVBL1 | RuvB-like 1 | DNA helicase activity; DNA binding; transcription factor activity; transcription cofactor activity | nucleobase, nucleoside, nucleotide and nucleic acid metabolic process; mesoderm development; embryonic development |
| MED18 | Mediator of RNA polymerase II transcription subunit 18 | nucleic acid binding; DNA polymerase processivity factor activity | nucleobase, nucleoside, nucleotide and nucleic acid metabolic process |
| KIF2A | Kinesin-like protein KIF2A | microtubule motor activity; structural constituent of cytoskeleton | intracellular protein transport; vesicle-mediated transport; mitosis; meiosis; cytokinesis; chromosome segregation; cellular component morphogenesis |
| MFHAS1 | Malignant fibrous histiocytoma-amplified sequence 1 | adenylate cyclase activity; receptor binding; kinase regulator activity | immune system process; cell surface receptor linked signal transduction; intracellular signaling cascade; nucleobase, nucleoside, nucleotide and nucleic acid metabolic process; signal transduction |
| **XRCC2** | DNA repair protein XRCC2 | hydrolase activity; DNA binding | immune system process; meiosis; nucleobase, nucleoside, nucleotide and nucleic acid metabolic process; meiosis; response to stress |
| STON2 | Stonin-2 | unclear molecular function | intracellular protein transport; endocytosis |
| CAMKK2 | Calcium/calmodulin-dependent protein kinase kinase 2 | kinase activity; structural constituent of cytoskeleton; cytoskeletal protein binding | immune system process; muscle contraction; neurological system process; mitosis; meiosis; intracellular signaling cascade; protein metabolic process; cytokinesis; signal transduction; cellular component morphogenesis; embryonic development; response to stress |
| PRKAG1 | 5'-AMP-activated protein kinase subunit gamma-1 | protein binding; kinase regulator activity | immune system process; intracellular signaling cascade; lipid metabolic process; protein metabolic process; signal transduction; response to stress |
| **GPI** | Glucose-6-phosphate isomerase | isomerase activity | carbohydrate metabolic process |
| SCP2 | Non-specific lipid-transfer protein | oxidoreductase activity | metabolic process |
| OXSR1 | Serine/threonine-protein kinase OSR1 | kinase activity | phosphate metabolic process; protein metabolic process |
| C11orf54 | Ester hydrolase C11orf54 | unclear molecular function | unclear biological function |
| XPO7 | Exportin-7 | unclear molecular function | intracellular protein transport |
| CYP4A11 | Cytochrome P450 4A11 | oxidoreductase activity | respiratory electron transport chain; lipid metabolic process |
| RNF41 | E3 ubiquitin-protein ligase NRDP1 | unclear molecular function | unclear biological function |
| DHRS2 | Dehydrogenase/reductase SDR family member 2; | oxidoreductase activity | visual perception; sensory perception; lipid metabolic process |
| DHRS4 | Dehydrogenase/reductase SDR family member 4 | oxidoreductase activity | visual perception; sensory perception; lipid metabolic process |
| **SCNM1** | Sodium channel modifier 1 | unclear molecular function | unclear biological function |
| PLEKHA2 | Pleckstrin homology domain-containing family A member 2 | unclear molecular function | unclear biological function |
| **MRPL40** | 39S ribosomal protein L40, mitochondrial | structural constituent of ribosome; nucleic acid binding | protein metabolic process |

## Table S4 - The list and characteristics of genes down-regulated in tumours group no. 1 in comparison to tumours group no. 3.

Symbol, name and characteristics of down-regulated genes in tumour group no. 1 in comparison to tumour group no. 3. The biological processes and molecular functions of the genes are based on the PANTHER database (www.pantherdb.org). Only significantly regulated genes were included in this table (p<0.01).

| **Gene symbol** | **Gene name** | **Molecular function** | **Biological process** |
| --- | --- | --- | --- |
| ZFAND6 | AN1-type zinc finger protein | nucleic acid binding; receptor binding | sensory perception of sound; sensory perception; respiratory electron transport chain; signal transduction |
| HIGD1B | HIG1 domain family member 1B | unclear molecular function | unclear molecular function |
| TIMP2 | Metalloproteinase inhibitor 2 | protein binding; peptidase inhibitor activity | protein metabolic process |
| HOXB7 | Homeobox protein Hox-B7 | DNA binding; transcription factor activity | female gamete generation; nucleobase, nucleoside, nucleotide and nucleic acid metabolic process; segment specification; ectoderm development; gut mesoderm development; embryonic development; skeletal system development; angiogenesis; nervous system development; muscle organ development |
| TRMT1 | N(2),N(2)-dimethylguanosine tRNA methyltransferase | methyltransferase activity; nucleic acid binding | nucleobase, nucleoside, nucleotide and nucleic acid metabolic process |
| RPL13 | 60S ribosomal protein L13 | structural constituent of ribosome; nucleic acid binding | protein metabolic process |
| LRGUK | Leucine-rich repeat and guanylate kinase domain-containing protein | structural constituent of cytoskeleton; protein binding; phosphatase regulator activity | mitosis; cell motion |
| BTBD6 | BTB/POZ domain-containing protein 6 | peptidase activity; structural constituent of cytoskeleton; DNA binding; cytoskeletal protein binding; transcription factor activity | nucleobase, nucleoside, nucleotide and nucleic acid metabolic process;protein metabolic process |
| FAM83E | Protein FAM83E | unclear molecular function | unclear molecular function |
| EIF4B | Eukaryotic translation initiation factor 4B | translation factor activity, nucleic acid binding; translation initiation factor activity | protein metabolic process |
| CALM3 | Calmodulin | calcium ion binding; calmodulin binding | cell cycle; intracellular signaling cascade; signal transduction |
| MORF4L1 | Mortality factor 4-like protein 1 | DNA binding; chromatin binding; transcription factor activity | nucleobase, nucleoside,  nucleotide and nucleic acid metabolic process |
| RNASEH1 | Ribonuclease H1 | RNA binding | nucleobase, nucleoside, nucleotide and nucleic acid metabolic process |
| ASTN2 | Astrotactin-2 | unclear molecular function | unclear molecular function |
| C6orf153 | UPF0399 protein C6orf153 | unclear molecular function | unclear molecular function |
| PFDN6 | Prefoldin subunit 6 | unclear molecular function | protein metabolic process |
| GAPDH | Glyceraldehyde-3-phosphate dehydrogenase | oxidoreductase activity | carbohydrate metabolic process |
| SLC41A2 | Solute carrier family 41 member 2 | cation transmembrane transporter activity | ion transport |
| KIF1B | Kinesin-like protein KIF1B | microtubule motor activity; structural constituent of cytoskeleton | intracellular protein transport; vesicle-mediated transport; mitosis; meiosis; cytokinesis; chromosome segregation; cellular component morphogenesis |
| UCKL1 | Uridine/cytidine kinase-like 1 | kinase activity; transferase activity, transferring glycosyl groups | carbohydrate metabolic process; nucleobase, nucleoside, nucleotide and nucleic acid metabolic process |
| CTNNA1 | Catenin alpha-1 | structural constituent of cytoskeleton; cytoskeletal protein binding | cellular component morphogenesis |
| TRAF3IP2 | Adapter protein CIKS | unclear molecular function | unclear molecular function |
| NFIX | Nuclear factor 1 X-type | DNA binding; transcription factor activity | nucleobase, nucleoside, nucleotide and nucleic acid metabolic process |
| SERPINF1 | Pigment epithelium-derived factor | protein binding; peptidase inhibitor activity | protein metabolic process |
| HBD | Hemoglobin subunit delta | unclear molecular function | blood circulation;transport |
| HLA-DQA2 | HLA class II histocompatibility antigen, DQ(6) alpha chain | unclear molecular function | unclear molecular function |
| HLA-DQB2 | HLA class II histocompatibility antigen, DX beta chain | unclear molecular function | antigen processing and presentation of peptide or polysaccharide antigen via MHC class II; cellular defense response |
| FAM172A | UPF0528 protein FAM172A | unclear molecular function | unclear molecular function |
| DHX40 | Probable ATP-dependent RNA helicase DHX40 | RNA helicase activity; nucleic acid binding | nucleobase, nucleoside, nucleotide and nucleic acid metabolic process |
| C3orf26 | Uncharacterized protein C3orf26 | RNA helicase activity; nucleic acid binding | nucleobase, nucleoside, nucleotide and nucleic acid metabolic process |
| MT2A | Metallothionein-2 | unclear molecular function | unclear molecular function |
| ALDH9A1 | 4-trimethylaminobutyraldehyde dehydrogenase | oxidoreductase activity | carbohydrate metabolic process;  nucleobase, nucleoside, nucleotide and nucleic acid metabolic process;  cellular amino acid and derivative metabolic process |
| CALM3 | Calmodulin | unclear molecular function | unclear molecular function |
| HMGN2 | Non-histone chromosomal protein HMG-17 | unclear molecular function | unclear molecular function |
| MRAS | Ras-related protein M-Ras | GTPase activity; protein binding | neurological system process; intracellular protein transport; endocytosis; cell surface receptor linked signal transduction; intracellular signaling cascade;  synaptic transmission; cell adhesion; signal transduction; cell-cell signaling |
| FLOT1 | Flotillin-1 | unclear molecular function | intracellular protein transport; vesicle-mediated transport |
| MORN5 | MORN repeat-containing protein 5 | kinase activity | cell surface receptor linked signal transduction; signal transduction |
| SLC22A9 | Solute carrier family 22 member 9 | ATPase activity, coupled to transmembrane movement of substances; ligase activity;  carbohydrate transmembrane transporter activity; cation transmembrane transporter activity | cation transport; anion transport; extracellular transport; carbohydrate transport; carbohydrate metabolic process |
| BXDC5 | Ribosome production factor 1 | RNA binding | nucleobase, nucleoside, nucleotide and nucleic acid metabolic process |
| PCGF2 | Polycomb group RING finger protein 2 | DNA binding; transcription factor activity | nucleobase, nucleoside, nucleotide and nucleic acid metabolic process |
| RIBC1 | RIB43A-like with coiled-coils protein 1 | structural constituent of cytoskeleton | unclear molecular function |
| HBG2 | Hemoglobin subunit gamma-2 | unclear molecular function | blood circulation;transport |
| TMEM120A | Transmembrane protein 120A | unclear molecular function | metabolic process |
| HLA-DQA1 | HLA class II histocompatibility antigen, DQ(5) alpha chain | unclear molecular function | antigen processing and presentation of peptide or polysaccharide antigen via MHC class II; cellular defense response |
| HLA-DQB1 | HLA class II histocompatibility antigen, DQ(3) beta chain | unclear molecular function | unclear molecular function |
| ACO2 | Aconitate hydratase, mitochondrial | hydro-lyase activity | tricarboxylic acid cycle; carbohydrate metabolic process; cellular amino acid and derivative metabolic process |
| CDC34 | Ubiquitin-conjugating enzyme E2 R1 | ubiquitin-protein ligase activity | apoptosis; mitosis; protein metabolic process; chromosome segregation |
| ZRSR2 | U2 small nuclear ribonucleoprotein auxiliary factor 35 kDa subunit-related protein 2 | RNA binding | nucleobase, nucleoside, nucleotide and nucleic acid metabolic process |
| PRKAR2B | cAMP-dependent protein kinase type II-beta regulatory subunit | protein binding; kinase regulator activity | signal transduction; protein metabolic process; signal transduction |
| HMGN2 | Non-histone chromosomal protein HMG-17 | nucleic acid binding; chromatin binding | cell cycle; nucleobase, nucleoside, nucleotide and nucleic acid metabolic process; |
| NDUFV3 | NADH dehydrogenase [ubiquinone] flavoprotein 3, mitochondrial | oxidoreductase activity | oxidative phosphorylation; respiratory electron transport chain; phosphate metabolic process |
| APOD | Apolipoprotein D | lipid transporter activity | lipid transport; lipid metabolic process |
| NUP88 | Nuclear pore complex protein Nup88 | transmembrane transporter activity | immune system process; intracellular protein transport; nuclear transport; response to stress |
| C1QB | Complement C1q subcomponent subunit B | receptor activity; extracellular matrix structural constituent; receptor binding | complement activation; carbohydrate transport; signal transduction; cell-cell signaling; cell adhesion; carbohydrate metabolic process; lipid metabolic process;  signal transduction; cellular component morphogenesis;  mesoderm development; skeletal system development; response to stimulus |
| CADPS2 | Calcium-dependent secretion activator 2 | calcium ion binding | unclear molecular function |
| VPS24 | Charged multivesicular body protein 3 | protein binding | intracellular protein transport; vesicle-mediated transport |
| KSR2 | Kinase suppressor of Ras 2 | kinase activity; transmembrane receptor protein serine/threonine kinase activity; transmembrane receptor protein tyrosine kinase activity; transmembrane receptor protein kinase activity | female gamete generation; immune system process; induction of apoptosis; negative regulation of apoptosis; cell cycle; cell surface receptor linked signal transduction; intracellular signaling cascade; protein metabolic process; cell cycle; signal transduction; cellular component morphogenesis; response to stress |
| BPI | Bactericidal permeability-increasing protein | unclear molecular function | macrophage activation; lipid transport; lipid metabolic process; response to stress; defense response to bacterium |
| NOL7 | Nucleolar protein 7 | unclear molecular function | unclear molecular function |
| MBOAT2 | Membrane-bound O-acyltransferase domain-containing protein 2 | acyltransferase activity | unclear molecular function |
| SERPINF2 | Alpha-2-antiplasmin | protein binding;peptidase inhibitor activity | protein metabolic process |
| WDR16 | WD repeat-containing protein 16 | unclear molecular function | unclear molecular function |
| MT1X | Metallothionein-1X | unclear molecular function | unclear molecular function |
| DPH3 | DPH3 homolog | unclear molecular function | unclear molecular function |
| SFRS9 | Splicing factor, arginine/serine-rich 9 | RNA splicing factor activity, transesterification mechanism; RNA binding | nucleobase, nucleoside, nucleotide and nucleic acid metabolic process |
| SLCO3A1 | Solute carrier organic anion transporter family member 3A1 | transmembrane transporter activity | anion transport |
| ODF3L1 | Outer dense fiber protein 3-like protein 1 | structural molecule activity | unclear molecular function |
| EPHB2 | Ephrin type-B receptor 2 | kinase activity; transmembrane receptor protein tyrosine kinase activity; transmembrane receptor protein kinase activity; receptor binding | female gamete generation; immune system process; negative regulation of apoptosis; cell cycle; cell surface receptor linked signal transduction; intracellular signaling cascade; cell-cell signaling; cell-cell adhesion; protein metabolic process; cell motion; cell cycle; signal transduction; cell-cell signaling; ectoderm development; mesoderm development; embryonic development; nervous system development |
| CBWD1 | COBW domain-containing protein 1 | unclear molecular function | vitamin biosynthetic process |
| KLC1 | Kinesin light chain 1 | structural constituent of cytoskeleton | intracellular protein transport; vesicle-mediated transport |
| NAALADL2 | Inactive N-acetylated-alpha-linked acidic dipeptidase-like protein 2 | peptidase activity | protein metabolic process |
| OTUB2 | Ubiquitin thioesterase OTUB2 | hydrolase activity | immune system process; protein metabolic process |
| C16orf33 | U11/U12 small nuclear ribonucleoprotein 25 kDa protein | unclear molecular function | unclear molecular function |
| DYNLRB1 | Dynein light chain roadblock-type 1 | structural constituent of cytoskeleton | cell motion |
| CHUK | Inhibitor of nuclear factor kappa-B kinase subunit alpha | kinase activity | immune response; intracellular signaling cascade; protein metabolic process; signal transduction; response to stimulus |
| MRC2 | C-type mannose receptor 2 | receptor activity | macrophage activation; intracellular protein transport; endocytosis |
| APOC1 | Apolipoprotein C-I | lipid transporter activity; transmembrane transporter activity | lipid transport; lipid metabolic process |
| NDUFA7 | NADH dehydrogenase [ubiquinone] 1 alpha subcomplex subunit 7 | oxidoreductase activity | unclear molecular function |
| ADCYAP1 | Pituitary adenylate cyclase-activating polypeptide 38 | receptor binding | cell surface receptor linked signal transduction; signal transduction |
| ELF3 | ETS-related transcription factor Elf-3 | DNA binding; receptor binding; transcription factor activity | B cell mediated immunity; cell cycle; cell surface receptor linked signal transduction; intracellular signaling cascade; nucleobase, nucleoside, nucleotide and nucleic acid metabolic process; cell cycle; signal transduction; endoderm development; mesoderm development; hemopoiesis; cellular defense response |
| HSDL2 | Hydroxysteroid dehydrogenase-like protein 2 | oxidoreductase activity | visual perception; sensory perception; lipid metabolic process |
| AUTS2 | Autism susceptibility gene 2 protein | unclear molecular function | unclear molecular function |
| HLA-DQB1 | HLA class II histocompatibility antigen, DQ(3) beta chain | unclear molecular function | antigen processing and presentation of peptide or polysaccharide antigen via MHC class II; cellular defense response |
| HLA-DRB1 | HLA class II histocompatibility antigen, DRB1-1 beta chain | unclear molecular function | antigen processing and presentation of peptide or polysaccharide antigen via MHC class II; cellular defense response |
| HLA-DRB5 | HLA class II histocompatibility antigen, DRB5 beta chain | unclear molecular function | antigen processing and presentation of peptide or polysaccharide antigen via MHC class II; cellular defense response |
| CALM3 | Calmodulin | unclear molecular function | unclear molecular function |
| CUX1 | Homeobox protein cut-like 1 | DNA binding; transcription factor activity | nucleobase, nucleoside, nucleotide and nucleic acid metabolic process |
| TFPI | Tissue factor pathway inhibitor | protein binding; peptidase inhibitor activity | immune system process; protein metabolic process; blood coagulation |
| SLC22A24 | Solute carrier family 22 member 24 | ATPase activity, coupled to transmembrane movement of substances; ligase activity; carbohydrate transmembrane transporter activity; cation transmembrane transporter activity | cation transport; anion transport; extracellular transport; carbohydrate transport; carbohydrate metabolic process |
| TMTC3 | Transmembrane and TPR repeat-containing protein 3 | transferase activity, transferring glycosyl groups | signal transduction; protein metabolic process; signal transduction |
| ARL4C | ADP-ribosylation factor-like protein 4C | GTPase activity; protein binding | intracellular protein transport; vesicle-mediated transport; signal transduction; signal transduction |
| SPTB | Spectrin beta chain, erythrocyte | unclear molecular function | unclear molecular function |
| TET3 | Protein TET3 | unclear molecular function | unclear molecular function |
| HLA-DPB1 | HLA class II histocompatibility antigen, DP(W2) beta chain | unclear molecular function | antigen processing and presentation of peptide or polysaccharide antigen via MHC class II; cellular defense response |
| HLA-DQB1 | HLA class II histocompatibility antigen, DQ(W1.1) beta chain | unclear molecular function | antigen processing and presentation of peptide or polysaccharide antigen via MHC class II; cellular defense response |
| MT1B | Metallothionein-1B | unclear molecular function | unclear molecular function |
| MT1H | Metallothionein-1H | unclear molecular function | unclear molecular function |
| KLHL10 | Kelch-like protein 10 | peptidase activity; structural constituent of cytoskeleton; DNA binding; cytoskeletal protein binding; transcription factor activity; transcription cofactor activity | neurological system process; nucleobase, nucleoside, nucleotide and nucleic acid metabolic process; protein metabolic process; cellular component morphogenesis |
| EIF3B | Eukaryotic translation initiation factor 3 subunit B | translation factor activity, nucleic acid binding; translation initiation factor activity | protein metabolic process |
| C1QC | Complement C1q subcomponent subunit C | receptor activity; extracellular matrix structural constituent; receptor binding | complement activation; carbohydrate transport; signal transduction; cell-cell signaling; cell adhesion; carbohydrate metabolic process; lipid metabolic process; signal transduction; cellular component morphogenesis; cellular component morphogenesis; mesoderm development; cellular component morphogenesis; skeletal system development; response to stimulus |
| KLHDC3 | Kelch domain-containing protein 3 | structural constituent of cytoskeleton; DNA binding; chromatin binding; protein binding; small GTPase regulator activity; transcription factor activity | spermatogenesis; immune system process; intracellular protein transport; vesicle-mediated transport; cell cycle; nitrogen compound metabolic process; nucleobase, nucleoside, nucleotide and nucleic acid metabolic process; protein metabolic process; cell cycle |
| NR2E1 | Nuclear receptor subfamily 2 group E member 1 | ligand-dependent nuclear receptor activity; DNA binding; transcription factor activity | visual perception; sensory perception; cell cycle; signal transduction; nucleobase, nucleoside, nucleotide and nucleic acid metabolic process; lipid metabolic process; cell cycle; signal transduction |
| FRAP1 | FKBP12-rapamycin complex-associated protein | kinase activity; nucleic acid binding | immune system process; induction of apoptosis; cell cycle; signal transduction; nucleobase, nucleoside, nucleotide and nucleic acid metabolic process; protein metabolic process; cell cycle; signal transduction; organelle organization; establishment or maintenance of chromatin architecture; response to stress |
| C9orf127 | Protein NGX6 | unclear molecular function | signal transduction; cell adhesion; signal transduction |
| U2AF2 | Splicing factor U2AF 65 kDa subunit | RNA splicing factor activity, transesterification mechanism; RNA binding | nucleobase, nucleoside, nucleotide and nucleic acid metabolic process |
| MEA1 | Male-enhanced antigen 1 | unclear molecular function | spermatogenesis |
| CA10 | Carbonic anhydrase-related protein 10 | hydro-lyase activity | metabolic process |
| HLA-DRB1 | HLA class II histocompatibility antigen, DRB1-10 beta chain | unclear molecular function | unclear molecular function |
| RRP12 | RRP12-like protein | unclear molecular function | unclear molecular function |
| NET1L_HUMAN | Netrin-1-like protein | unclear molecular function | unclear molecular function |
| CUL1 | Cullin-1 | ubiquitin-protein ligase activity | induction of apoptosis; mitosis; protein metabolic process |
| MYBPC1 | Myosin-binding protein C, slow-type | kinase activity; structural constituent of cytoskeleton; protein binding; small GTPase regulator activity; guanyl-nucleotide exchange factor activity | muscle contraction; intracellular protein transport; endocytosis; signal transduction; cell adhesion; protein metabolic process; cell motion; signal transduction; cell adhesion; cellular component morphogenesis; cellular component morphogenesis; mesoderm development; cellular component morphogenesis; muscle organ development |
| CBWD2 | COBW domain-containing protein 2 | unclear molecular function | vitamin biosynthetic process |
| TXN | Thioredoxin | oxidoreductase activity | immune system process; respiratory electron transport chain; apoptosis; meiosis; intracellular signaling cascade; sulfur metabolic process; signal transduction; response to stress |
| PFDN6 | Prefoldin subunit 6 | unclear molecular function | unclear molecular function |
| PFKP | 6-phosphofructokinase type C | kinase activity | carbohydrate metabolic process |
| VTCN1 | V-set domain-containing T-cell activation inhibitor 1 | Unclear molecular function | immune system process; cellular defense response |
| ORC6L | Origin recognition complex subunit 6 | DNA binding | cell cycle; nucleobase, nucleoside, nucleotide and nucleic acid metabolic process; cell cycle |
| TLK2 | Serine/threonine-protein kinase tousled-like 2 | kinase activity | cell cycle; intracellular signaling cascade; nucleobase, nucleoside, nucleotide and nucleic acid metabolic process; protein metabolic process; cell cycle; signal transduction |
| RNPEP | Aminopeptidase B | peptidase activity | immune system process; lipid metabolic process; protein metabolic process |
| CERK | Ceramide kinase | kinase activity | cell surface receptor linked signal transduction; signal transduction |
| VBP1 | Prefoldin subunit 3 | unclear molecular function | protein metabolic process |
| HLA-DPB1 | HLA class II histocompatibility antigen, DP(W4) beta chain | unclear molecular function | antigen processing and presentation of peptide or polysaccharide antigen via MHC class II; cellular defense response |
| HLA-DPB1 | HLA class II histocompatibility antigen, SB beta chain | unclear molecular function | antigen processing and presentation of peptide or polysaccharide antigen via MHC class II; cellular defense response |
| HLA-DRB1 | HLA class II histocompatibility antigen, DRB1-10 beta chain | unclear molecular function | antigen processing and presentation of peptide or polysaccharide antigen via MHC class II; cellular defense response |
| HLA-DQA2 | HLA class II histocompatibility antigen, DQ(6) alpha chain | unclear molecular function | antigen processing and presentation of peptide or polysaccharide antigen via MHC class II; cellular defense response |
| HN1 | Hematological and neurological expressed 1 protein | unclear molecular function | unclear molecular function |
| VWA2 | von Willebrand factor A domain-containing protein 2 | extracellular matrix structural constituent | immune system process; sensory perception of sound; sensory perception; signal transduction; cell-cell adhesion; cellular component morphogenesis; mesoderm development; skeletal system development; blood coagulation |
| MT1A | Metallothionein-1A | unclear molecular function | unclear molecular function |
| MT1G | Metallothionein-1G | unclear molecular function | unclear molecular function |
| MAD1L1 | Mitotic spindle assembly checkpoint protein MAD1 | unclear molecular function | mitosis |
| TOE1 | Target of EGR1 protein 1 | RNA binding | nucleobase, nucleoside, nucleotide and nucleic acid metabolic process |
| TREX1 | Three prime repair exonuclease 1 | hydrolase activity, acting on ester bonds; nucleic acid binding | unclear molecular function |
| ANKHD1 | Ankyrin repeat and KH domain-containing protein 1 | unclear molecular function | unclear molecular function |
| FLOT1 | Flotillin-1 | unclear molecular function | unclear molecular function |
| FLOT1 | Flotillin-1 | unclear molecular function | unclear molecular function |
| CBWD3 | COBW domain-containing protein 3 | unclear molecular function | vitamin biosynthetic process |
| CRIP1 | Cysteine-rich protein 1 | hydrolase activity, acting on ester bonds; structural constituent of cytoskeleton; DNA binding; cytoskeletal protein binding; transcription factor activity; transcription cofactor activity | immune system process; muscle contraction; negative regulation of apoptosis; signal transduction; nucleobase, nucleoside, nucleotide and nucleic acid metabolic process; cell motion; signal transduction; cellular component morphogenesis; cellular component morphogenesis; ectoderm development; mesoderm development; cellular component morphogenesis; embryonic development; skeletal system development; nervous system development; heart development; muscle organ development; cellular defense response |
| TRAPPC2P | Trafficking protein particle complex subunit 2 | DNA binding; transcription factor activity | unclear molecular function |

## Table S5 - The list and characteristics of genes up-regulated in tumours group no. 2 in comparison to tumours group no. 3.

Symbol, name and characteristics of up-regulated genes in tumour group no. 2 in comparison to tumour group no. 3. The biological processes and molecular functions of the genes are based on the PANTHER database (www.pantherdb.org). Only significantly regulated genes were included in this table (p<0.01).

| **Gene symbol** | **Gene name** | **Molecular function** | **Biological process** |
| --- | --- | --- | --- |
| FAM120A | Constitutive coactivator of PPAR-gamma-like protein 1 | unclear molecular function | unclear biological process |
| PPTC7 | Protein phosphatase PTC7 homolog | hydrolase activity, acting on ester bonds; phosphatase activity | protein metabolic process |
| XRN2 | 5'-3' exoribonuclease 2 | hydrolase activity, acting on ester bonds; nucleic acid binding | nucleobase, nucleoside, nucleotide and nucleic acid metabolic process |
| ARPP21 | cAMP-regulated phosphoprotein 21 | unclear molecular function | nucleobase, nucleoside, nucleotide and nucleic acid metabolic process |
| GALNT11 | Polypeptide N-acetylgalactosaminyltransferase 11 | transferase activity, transferring glycosyl groups | carbohydrate metabolic process; protein metabolic process |
| DYNC1I2 | Cytoplasmic dynein 1 intermediate chain 2 | structural constituent of cytoskeleton | intracellular protein transport; vesicle-mediated transport; cell cycle; nucleobase, nucleoside, nucleotide and nucleic acid metabolic process; cell cycle; cellular component morphogenesis; RNA localization |
| DPYSL3 | Dihydropyrimidinase-related protein 3 | hydrolase activity | nucleobase, nucleoside, nucleotide and nucleic acid metabolic process |
| DPYSL2 | Dihydropyrimidinase-related protein 2 | hydrolase activity | nucleobase, nucleoside, nucleotide and nucleic acid metabolic process |
| MDGA2 | MAM domain-containing glycosylphosphatidylinositol anchor protein 2 | unclear molecular function | unclear biological process |
| YAP1 | 65 kDa Yes-associated protein | DNA binding; protein binding; kinase regulator activity; transcription factor activity; transcription cofactor activity | signal transduction; nucleobase, nucleoside, nucleotide and nucleic acid metabolic process;signal transduction |
| ZBED5 | Zinc finger BED domain-containing protein 5 | DNA binding; protein binding; kinase inhibitor activity; kinase regulator activity; transcription factor activity | nucleobase, nucleoside, nucleotide and nucleic acid metabolic process |
| ST3GAL2 | CMP-N-acetylneuraminate-beta-galactosamide-alpha-2,3-sialyltransferase | transferase activity, transferring glycosyl groups | carbohydrate metabolic process; lipid metabolic process; protein metabolic process |
| MADD | MAP kinase-activating death domain protein | protein binding; small GTPase regulator activity; guanyl-nucleotide exchange factor activity | neurological system process; induction of apoptosis;  intracellular signaling cascade; synaptic transmission;  signal transduction; cell-cell signaling; ectoderm development; nervous system development |
| FTH1 | Ferritin heavy chain | unclear molecular function | cation transport |
| SDHA | Succinate dehydrogenase [ubiquinone] flavoprotein subunit, mitochondrial | oxidoreductase activity | oxidative phosphorylation; respiratory electron transport chain; carbohydrate metabolic process |
| C14orf1 | Probable ergosterol biosynthetic protein 28 | unclear molecular function | unclear biological process |
| ACSS3 | Acyl-CoA synthetase short-chain family member 3, mitochondrial | ligase activity | immune system process; coenzyme metabolic process; lipid metabolic process |
| BIRC5 | Baculoviral IAP repeat-containing protein 5 | unclear molecular function | unclear biological process |
| TIPRL | TIP41-like protein | unclear molecular function | unclear biological process |
| HOOK3 | Protein Hook homolog 3 | protein binding; kinase regulator activity | intracellular protein transport; cell surface receptor linked signal transduction; intracellular signaling cascade; signal transduction |
| SLC39A11 | Zinc transporter ZIP11 | unclear molecular function | unclear biologicall process |
| PSMC3 | 26S protease regulatory subunit 6A | hydrolase activity | protein metabolic process |
| PAN2 | PAB-dependent poly(A)-specific ribonuclease subunit 2 | peptidase activity; hydrolase activity, acting on ester bonds; nucleic acid binding | nucleobase, nucleoside, nucleotide and nucleic acid metabolic process;protein metabolic process |
| JTB | Protein JTB | unclear molecular function | unclear biological process |
| NPHP3 | Nephrocystin-3 | structural constituent of cytoskeleton | intracellular protein transport; vesicle-mediated transport |
| SMARCA1 | Probable global transcription activator SNF2L1 | DNA helicase activity; nucleic acid binding | nucleobase, nucleoside, nucleotide and nucleic acid metabolic process; organelle organization; establishment or maintenance of chromatin architecture |
| EPHA4 | Ephrin type-A receptor 4 | kinase activity; transmembrane receptor protein tyrosine kinase activity; transmembrane receptor protein kinase activity; receptor binding | female gamete generation; immune system process; negative regulation of apoptosis; cell cycle; cell surface receptor linked signal transduction; intracellular signaling cascade; cell-cell signaling; cell-cell adhesion; protein metabolic process; cell motion; cell cycle; signal transduction; ectoderm development; mesoderm development; embryonic development; nervous system development |
| CCDC28A | Coiled-coil domain-containing protein 28A | receptor activity | unclear biological process |
| CSDE1 | Cold shock domain-containing protein E1 | unclear molecular function | unclear biological process |
| MYCN | N-myc proto-oncogene protein | DNA binding; transcription factor activity | induction of apoptosis; cell cycle;  intracellular signaling cascade; nucleobase, nucleoside, nucleotide and nucleic acid metabolic process; cell cycle; signal transduction |
| ZNF536 | Zinc finger protein 536 | DNA binding; transcription factor activity | nucleobase, nucleoside, nucleotide and nucleic acid metabolic process |
| LRRC8A | Leucine-rich repeat-containing protein 8A | adenylate cyclase activity; receptor binding; kinase regulator activity | immune system process; cell surface receptor linked signal transduction; intracellular signaling cascade;  nucleobase, nucleoside, nucleotide and nucleic acid metabolic process; signal transduction |
| RPS8 | 40S ribosomal protein S8 | structural constituent of ribosome;nucleic acid binding | protein metabolic process |
| REN | Renin | peptidase activity | antigen processing and presentation of peptide or polysaccharide antigen via MHC class II; protein metabolic process; cellular defense response |
| ATP2A2 | Sarcoplasmic/endoplasmic reticulum calcium ATPase 2 | hydrolase activity; cation transmembrane transporter activity; ion channel activity | cation transport; lipid transport; lipid metabolic process; cellular calcium ion homeostasis |
| RTF1 | RNA polymerase-associated protein RTF1 homolog | unclear molecular function | unclear biological process |
| MYH7 | Myosin-7 | motor activity; structural constituent of cytoskeleton; protein binding; small GTPase regulator activity | muscle contraction; sensory perception of sound; sensory perception; intracellular protein transport; vesicle-mediated transport; mitosis; intracellular signaling cascade; cytokinesis; cell motion; signal transduction; cellular component morphogenesis; cellular component morphogenesis; mesoderm development; muscle organ development |
| SAP30L | Histone deacetylase complex subunit SAP30L | nucleic acid binding; chromatin binding | nucleobase, nucleoside, nucleotide and nucleic acid metabolic process |
| PRDX1 | Peroxiredoxin-1 | oxidoreductase activity; peroxidase activity | immune system process; oxygen and reactive oxygen species metabolic process |
| PLEKHM3 | Pleckstrin homology domain-containing family M member 3 | unclear molecular function | unclear biological process |
| NOS2 | Nitric oxide synthase, inducible | oxidoreductase activity; calcium ion binding; calmodulin binding | respiratory electron transport chain; intracellular signaling cascade; sulfur metabolic process  nitric oxide biosynthetic process; cellular amino acid and derivative metabolic process; signal transduction |
| DENND1A | DENN domain-containing protein 1A | unclear molecular function | unclear biological process |
| DNAJB6 | DnaJ homolog subfamily B member 6 | unclear molecular function | immune system process; protein metabolic process; response to stress |
| ITIH3 | Inter-alpha-trypsin inhibitor heavy chain H3 | protein binding; peptidase inhibitor activity | protein metabolic process |
| COMT | Catechol O-methyltransferase | methyltransferase activity | neurological system process; metabolic process |
| SYNE2 | Nesprin-2 | unclear molecular function | unclear biological process |
| HBE1 | Hemoglobin subunit epsilon | unclear molecular function | blood circulation; transport |
| E2F3 | Transcription factor E2F3 | DNA binding; transcription factor activity | cell cycle; signal transduction; nucleobase, nucleoside, nucleotide and nucleic acid metabolic process; cell cycle |
| PCDH9 | Protocadherin-9 | G-protein coupled receptor activity; calcium ion binding | visual perception; sensory perception of sound; sensory perception; cell surface receptor linked signal transduction; cell-cell adhesion; cell motion;  signal transduction; cell-cell adhesion; cellular component morphogenesis; ectoderm development; mesoderm development; embryonic development; nervous system development; heart development; muscle organ development |
| ENOX2 | Protein disulfide-thiol oxidoreductase | oxidoreductase activity; hydrolase activity, acting on ester bonds; nucleic acid binding | respiratory electron transport chain; metabolic process |
| PTGES | Prostaglandin E synthase | transferase activity; isomerase activity | immune system process; lipid metabolic process; protein metabolic process; response to toxin |
| ACAD9 | Acyl-CoA dehydrogenase family member 9, mitochondrial | oxidoreductase activity; transferase activity | respiratory electron transport chain;  acyl-CoA metabolic process; nitrogen compound metabolic process; lipid metabolic process |
| ITIH4 | 35 kDa inter-alpha-trypsin inhibitor heavy chain H4 | protein binding; peptidase inhibitor activity | protein metabolic process |
| ALDOC | Fructose-bisphosphate aldolase C | lyase activity | carbohydrate metabolic process |
| CDKN1B | Cyclin-dependent kinase inhibitor 1B | protein binding; kinase inhibitor activity; kinase regulator activity | cell cycle; |
| CREB3L4 | Processed cAMP-responsive element-binding protein 3-like protein 4 | DNA binding; transcription factor activity | immune system process; neurological system process;signal transduction; nucleobase, nucleoside, nucleotide and nucleic acid metabolic process; signal transduction; ectoderm development; nervous system development; response to stress |

## Table S6 - The list and characteristics of genes down-regulated in tumours group no. 2 in comparison to tumours group no. 3.

Symbol, name and characteristics of down-regulated genes in tumour group no. 2 in comparison to tumour group no. 3. The biological processes and molecular functions of the genes are based on the PANTHER database (www.pantherdb.org). Only significantly regulated genes were included in this table (p<0.01).

| **Gene symbol** | **Gene name** | **Molecular function** | **Biological process** |
| --- | --- | --- | --- |
| ATP5J | ATP synthase-coupling factor 6, mitochondrial | catalytic activity; cation transmembrane transporter activity; hydrogen ion transmembrane transporter activity | oxidative phosphorylation; respiratory electron transport chain; cation transport |
| AP1S2 | AP-1 complex subunit sigma-2 | unclear molecular function | intracellular protein transport; endocytosis |
| COX4NB | Neighbor of COX4 | unclear molecular function | unclear biological process |
| CAP1 | Adenylyl cyclase-associated protein 1 | structural constituent of cytoskeleton | intracellular signaling cascade; signal transduction |
| ZFAND6 | AN1-type zinc finger protein 6 | nucleic acid binding; receptor binding | sensory perception of sound; sensory perception; respiratory electron transport chain; signal transduction |
| EPX | Eosinophil peroxidase heavy chain | oxidoreductase activity; peroxidase activity | immune response; oxygen and reactive oxygen species metabolic process; response to stimulus |
| GPM6B | Neuronal membrane glycoprotein M6-b | structural constituent of myelin sheath | neurological system process; cellular component morphogenesis; ectoderm development; cellular component morphogenesis; nervous system development |
| ARHGEF10L | Rho guanine nucleotide exchange factor 10-like protein | peptidase activity | protein metabolic process |
| TINP1 | Ribosome biogenesis protein NSA2 homolog | unclear molecular function | unclear biological process |
| CCNYL1 | Cyclin-Y-like protein 1 | unclear molecular function | unclear biological process |
| FECH | Ferrochelatase, mitochondrial | lyase activity | porphyrin metabolic process |
| PRKAR2B | cAMP-dependent protein kinase type II-beta regulatory subunit | protein binding; kinase regulator activity | signal transduction; protein metabolic process; signal transduction |
| CDC34 | Ubiquitin-conjugating enzyme E2 R1 | ubiquitin-protein ligase activity | apoptosis; mitosis; protein metabolic process; chromosome segregation |
| DGCR6 | Protein DGCR6 | unclear molecular function | unclear biological process |
| TSSC4 | Protein TSSC4 | unclear molecular function | unclear biological process |
| TIMM10 | Mitochondrial import inner membrane translocase subunit Tim10 | unclear molecular function | intracellular protein transport; organelle organization; mitochondrion organization |
| MTMR15 | Coiled-coil domain-containing protein MTMR15 | unclear molecular function | unclear biological process |
| ISYNA1 | Inositol-3-phosphate synthase 1 | isomerase activity | lipid metabolic process |
| DGCR6L | Protein DGCR6L | unclear molecular function | unclear biological process |
| GSTA3 | Glutathione S-transferase A3 | transferase activity | immune system process; muscle contraction; lipid metabolic process; response to toxin |
| NAMPT | Nicotinamide phosphoribosyltransferase | receptor binding | immune system process; signal transduction; ell-cell signaling |
| CUGBP1 | CUG-BP- and ETR-3-like factor 1 | RNA splicing factor activity, transesterification mechanism; RNA binding | signal transduction; nucleobase, nucleoside, nucleotide and nucleic acid metabolic process; protein metabolic process;signal transduction; ectoderm development; nervous system development |
| WDR19 | WD repeat-containing protein 19 | unclear molecular function | unclear biological process |
| TESSP2 | Putative testis serine protease 2 | peptidase activity; calcium ion binding; receptor binding; calmodulin binding; calcium-dependent phospholipid binding; peptidase inhibitor activity | female gamete generation; fertilization; complement activation; blood circulation; lipid transport; apoptosis; lipid metabolic process; protein metabolic process; mesoderm development; angiogenesis; blood coagulation |
| MBOAT2 | Membrane-bound O-acyltransferase domain-containing protein 2 | acyltransferase activity | unclear biological process |
| SLC12A3 | Solute carrier family 12 member 3 | cation transmembrane transporter activity | cation transport |
| GSTA2 | Glutathione S-transferase A2 | transferase activity | immune system process; muscle contraction; lipid metabolic process; response to toxin |
| CD164 | Putative mucin core protein 24 | unclear molecular function | signal transduction; cell-cell adhesion |
| AFG3L2 | AFG3-like protein 2 | peptidase activity | intracellular protein transport; protein metabolic process; organelle organization; mitochondrion organization |
| RAPGEF5 | Rap guanine nucleotide exchange factor 5 | protein binding; small GTPase regulator activity; guanyl-nucleotide exchange factor activity | mitosis; cell surface receptor linked signal transduction; intracellular signaling cascade; mitosis; signal transduction |
| SAR1A | GTP-binding protein SAR1a | GTPase activity; protein binding | intracellular protein transport; vesicle-mediated transport; signal transduction; signal transduction |
| PTPRF | Receptor-type tyrosine-protein phosphatase F | hydrolase activity, acting on ester bonds; phosphatase activity; receptor activity | immune system process; intracellular protein transport; mitosis; cell surface receptor linked signal transduction; intracellular signaling cascade; cell-matrix adhesion; cell-cell adhesion; protein metabolic process; cytokinesis; cell motion; mitosis; signal transduction; nervous system development; cellular glucose homeostasis |
| GSTA5 | Glutathione S-transferase A5 | transferase activity | immune system process; muscle contraction; lipid metabolic process; response to toxin |
| JAZF1 | Juxtaposed with another zinc finger protein 1 | unclear molecular function | unclear biological process |
| GSTA4 | Glutathione S-transferase A4 | transferase activity | immune system process; muscle contraction; lipid metabolic process; response to toxin |
| SIPA1L2 | Signal-induced proliferation-associated 1-like protein 2 | protein binding; small GTPase regulator activity | cell adhesion; cell adhesion |
| SPG11 | Spatacsin | unclear molecular function | unclear biological process |
| GSTA1 | Glutathione S-transferase A1 | transferase activity | immune system process; muscle contraction; lipid metabolic process; response to toxin |
| MINK1 | Misshapen-like kinase 1 | kinase activity | phosphate metabolic process; protein metabolic process |
| TXN | Thioredoxin | oxidoreductase activity | immune system process; respiratory electron transport chain; apoptosis; meiosis; intracellular signaling cascade; sulfur metabolic process; signal transduction; response to stress |
| LAMP1 | Lysosome-associated membrane glycoprotein 1 | unclear molecular function | lysosomal transport; intracellular protein transport; protein metabolic process |
| CTCF | Transcriptional repressor CTCF | DNA binding; transcription factor activity | nucleobase, nucleoside, nucleotide and nucleic acid metabolic process |
| POLR2G | DNA-directed RNA polymerase II subunit RPB7 | DNA-directed RNA polymerase activity; nucleotidyltransferase activity; DNA binding; transcription factor activity | nucleobase, nucleoside, nucleotide and nucleic acid metabolic process |
| CAPZB | F-actin-capping protein subunit beta | structural constituent of cytoskeleton; cytoskeletal protein binding | cellular component morphogenesis |
| RARB | Retinoic acid receptor beta | ligand-dependent nuclear receptor activity; DNA binding; transcription factor activity | signal transduction; nucleobase, nucleoside, nucleotide and nucleic acid metabolic process; signal transduction; embryonic development |
| ANKRD10 | Ankyrin repeat domain-containing protein 10 | unclear molecular function | unclear biological process |
| WDR74 | WD repeat-containing protein 74 | calcium ion binding; calmodulin binding; calcium-dependent phospholipid binding | unclear biological process |
